# Supplementary material for: Oxidative Stress Triggers Selective tRNA Retrograde Transport in Human Cells during the Integrated Stress Response
Source: Cell Rep. 2019 Mar 19;26(12):3416–3428.e5. doi: 10.1016/j.celrep.2019.02.077 (PMC6426654; doi:10.1016/j.celrep.2019.02.077)
Supplement: Document S1. Figures S1–S7 and Table S1 [file mmc1.pdf]

Cell Reports, Volume *26*

Supplemental Information

Oxidative Stress Triggers Selective tRNA Retrograde Transport in Human Cells during the Integrated Stress Response

Hagen Schwenzer, Frank Jühling, Alexander Chu, Laura J. Pallett, Thomas F. Baumert, Mala Maini, and Ariberto Fassati

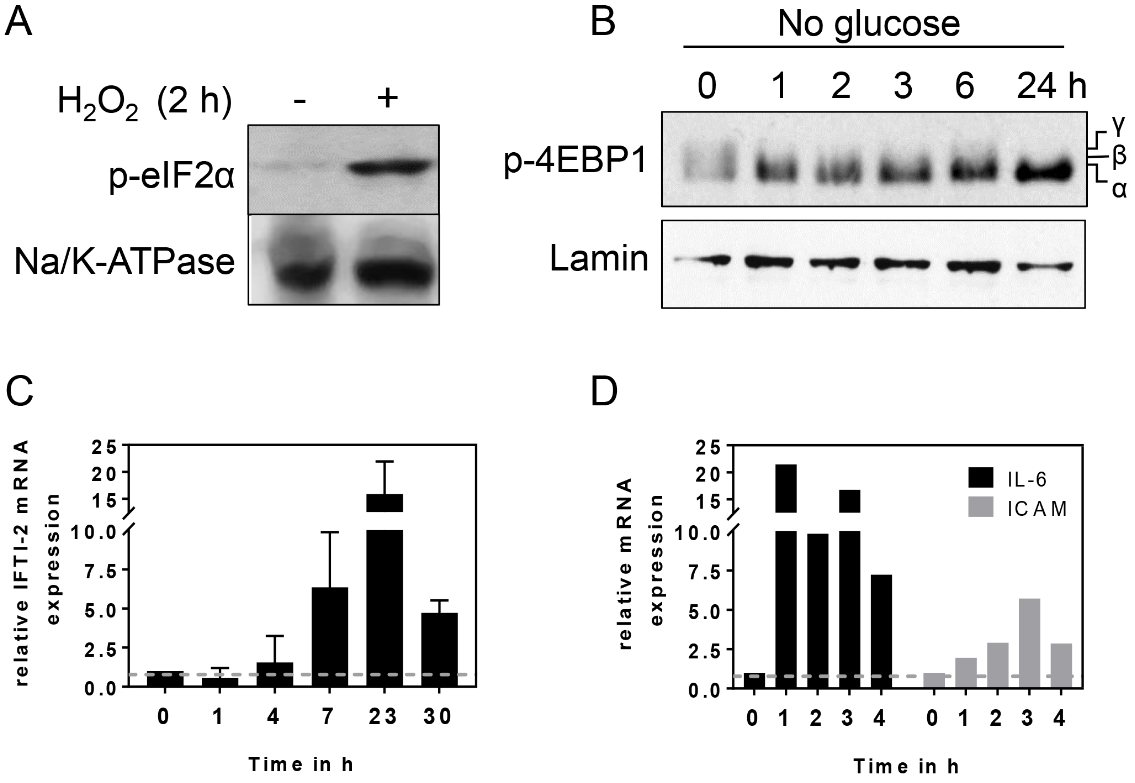

Figure S1 (related to Figure 1). Detection of cellular stress markers.

(A) Western blot to detect S51 phosphorylated eIF2α (p-eIF2α) after treatment with 5 mM H_2O_2 for 2 hours. (B) Western blot to detect changes in 4EBP1 phosphorylation at different time points in response to glucose deprivation, lamin was used as a loading control. (C) RT qPCR to measure IFTI-2 mRNA levels in response to IFNα (1.25 x10^4 U/ml). (D) Same as (C) to measure IL-6 and ICAM mRNA levels upon treatment with TNFα (1ng/ml). Values are expressed relative to GAPDH mRNA as mean ± SEM.

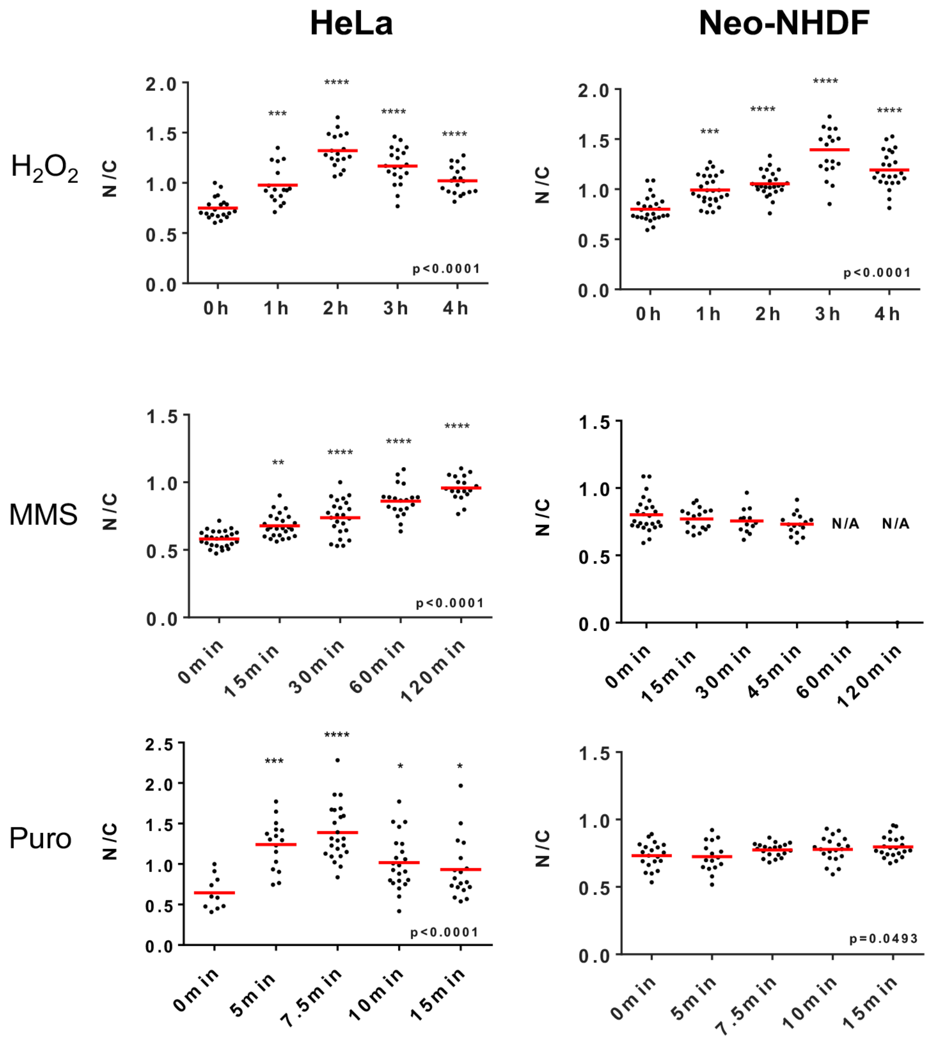

Figure S2 (related to Figure 2). Kinetics of tRNA retrograde transport in HeLa and NHDF cells.

Cells were treated with 5 mM H_2O_2 or 5 mM MMS or 3 mM puromycin for the indicated time points and analyzed by tFISH and confocal microscopy. ImageJ software was used to quantify the fluorescent signal in the nucleus (N) and cytoplasm (C) and calculate the N/C ratio. Cells were counted from at least 5 randomly chosen images. Each dot corresponds to one cell. Red lines indicate the mean value. Graphs show data from one representative experiment ($n \geq 3$). One-way Anova (Dunnett's multiple comparisons test) was used to calculate statistical significance *p=0.05; **p<0.05; ****p<0.0001. N/A, not enough cells for quantification due to toxicity.

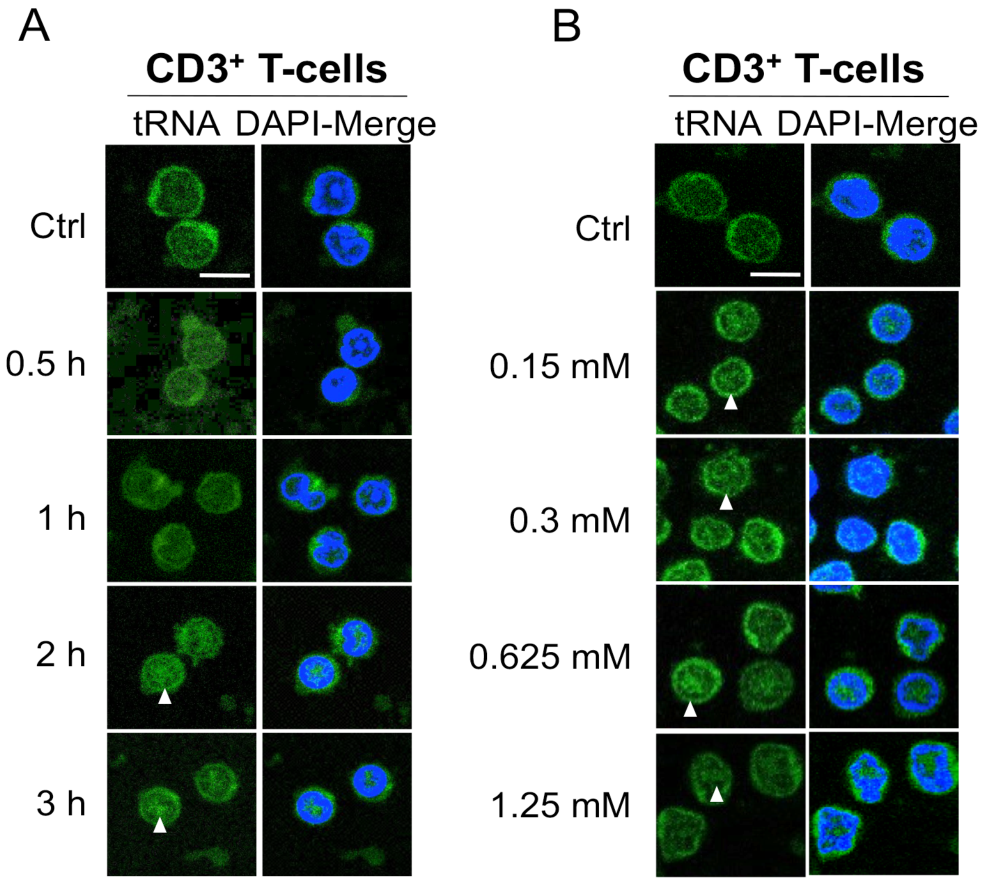

Figure S3 (related to Figure 2). Time and dose dependent induction of tRNA retrograde transport in primary CD3+ T-cells.

(A) Magnetic-bead purified T-cells were exposed to 0.6 mM H_2O_2 and incubated for the indicated period of time before analysis by tFISH. Representative confocal microscopy images from one donor are shown. Similar results were obtained with a second donor. (B) T-cells were exposed for 2 hours to the indicated concentrations of H_2O_2 and analysed by tFISH and confocal microscopy. Representative confocal microscopy images are shown. Increasing concentrations of H_2O_2 led to an increase of the tRNA signal in the nucleus. The signal intensity rose until plateau was reached, afterwards the overall fluorescence intensity decreased and frequent cell death was observed. Representative images from one donor are shown. Similar results were obtained with a second donor. Arrowheads point to intra-nuclear signal. Scale bar = 10µm.

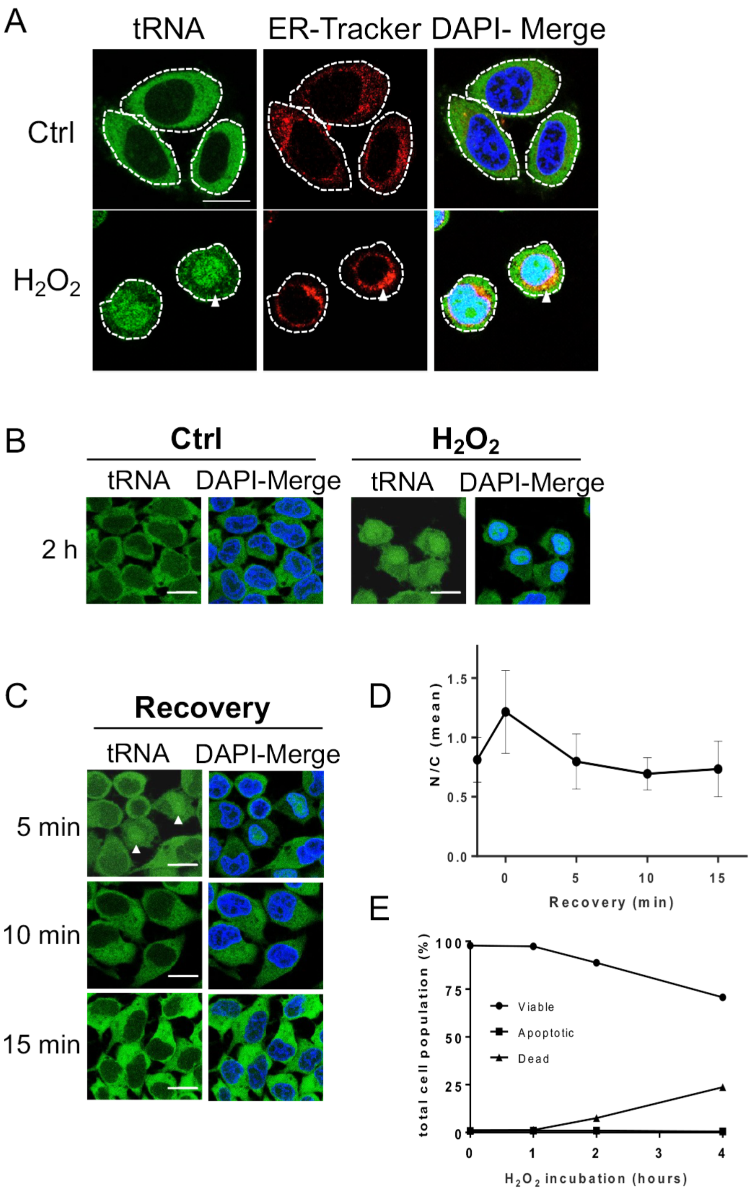

Figure S4 (related to Figure 3). tRNA retrograde transport depletes the ER and is reversible.

(A) Cells were pre-incubated with ActD for 2 hours then 5 mM H_2O_2 was added for 2 hours. Cells were incubated with ER Tracker^{TM} Red (Molecular Probes) and analysed by tFISH and confocal microscopy. Scale bar = 10µm. Arrowheads indicate the ER. (B-C) Induction of retrograde tRNA transport by H_2O_2 is reversible. Cells were exposed to 5 mM H_2O_2 for 2 hours before media was replaced without H_2O_2 (Recovery) and cells were analyzed by tFISH at the indicated time points. Representative confocal microscopy images are shown (n =3), scale bar = 20µm. Arrow heads indicate nuclei with a positive tFISH signal. (D) The tFISH fluorescent signal intensity in the nucleus and cytoplasm was quantified by ImageJ, the ratio nuclear:cytoplasmic signal (N/C) was calculated and values were plotted as a function of time. Graph shows average values ± SD from three independent experiments. (E) Flow cytometry analysis of apoptotic (annexin stain) and dead (PI stain) cells within the total population of cells treated with 5 mM H_2O_2.

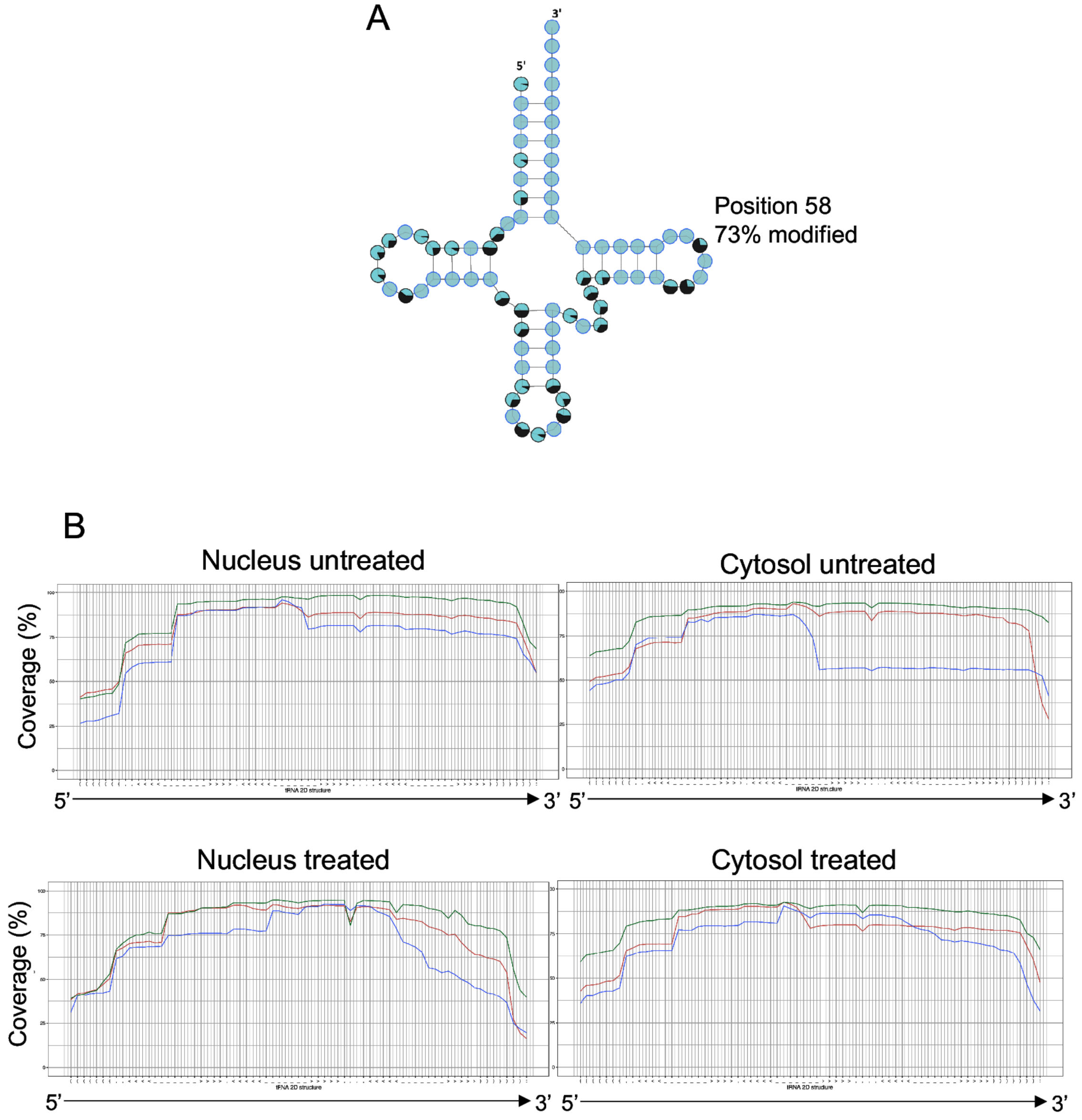

Figure S5 (related to Figure 4). Reduced 5' end coverage did not affect 3' mapping of tRNAs.

(A) Illustration of the secondary structure of the human consensus tRNA model. Each circle represents one nucleotide. Filling of circles represents a pie chart indicating the frequency of known modification at the particular position. Data has been extracted from MODOMICS (http://modomics.genesilico.pl/). (B) Alignment of sequences based on covariance model build from ~1000 tRNAs extracted from RFAM. Reads for each position have been counted and divided by total reads. Lines represent the relative coverage normalised to total tRNA reads. Each line represents one biological replicate. Treated, cells exposed for 5 mM H_2O_2 for 2 hours in the presence of ActD; untreated, cells exposed to ActD only for 2 hours.

A

	nucleus treated			cytosol treated			nucleus control			cytosol control		
**Replicates**	**1**	**2**	**3**	**1**	**2**	**3**	**1**	**2**	**3**	**1**	**2**	**3**
**total reads (in Mio)**	11.44	11.35	11.29	15.05	13.32	9.70	7.25	8.38	6.46	10.83	14.01	7.00
**mapped reads (%)**	66.76	46.93	5.04	58.34	13.07	0.33	73.94	88.60	3.21	79.89	27.78	2.26
mRNA (%)	27.61	28.94	28.95	9.22	13.56	22.66	31.95	35.34	36.66	8.79	11.27	14.89
ncRNA (%)	71.91	70.55	67.75	82.79	83.14	69.78	66.58	64.13	62.15	82.80	83.45	81.14
tRNA (%)	2.14	2.21	17.18	26.01	11.92	33.40	5.64	1.94	5.12	29.08	17.19	17.24

B

Nucleus		Cytosol	
**Ensembl ID**	**Reads ($x10^3$)**	**Ensembl ID**	**Reads ($x10^3$)**
RNU2-2P	869	MT-TV	418
SNORA51	258	CTD-2651B20.6	266
RNU5D-1	189	CTD-2651B20.7	265
SNORA2B	132	MT-TQ	261
RNU2-59P	120	MT-TN	236
SNORA28	118	RP11-473M20.16	233
SNORD14B	114	MT-TI	225
SNORA77	114	MT-TR	195
SNORA6	110	MT-TE	192
SNORA45	102	MT-TP	168

C

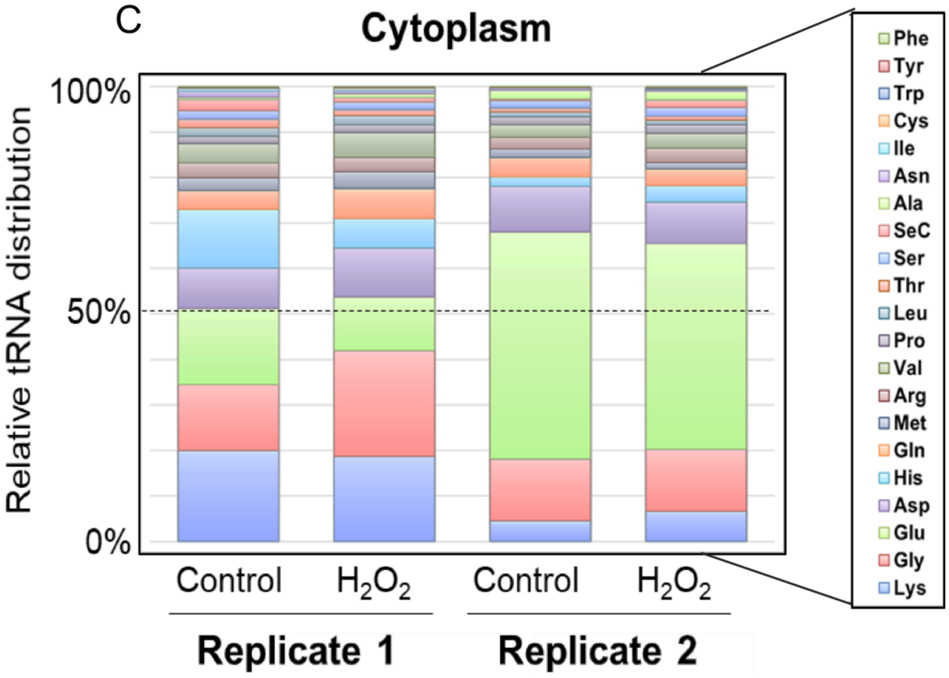

Figure S6 (related to Figure 4). Assessment of the quality of the cellular fractionation and RNAseq. HeLa cells were treated with 5 mM H_2O_2 for 2 hours in the presence of ActD and fractionated into nuclear and cytosolic fractions. RNA was extracted from each fraction and used for RNA-Seq. (A) Summary of the mapped RNA reads in each replicate and their distribution in each fraction. (B) Top 10 reads detected in the nuclear and

cytosolic fraction of one representative control replicate. Treated, cells exposed for 5 mM H_2O_2 for 2 hours in the presence of ActD; untreated, cells exposed to ActD only for 2 hours. (C) Total RNA reads were mapped to 323 unique tRNA genes and all reads mapping to the same tRNA species were counted, combined and their relative distribution calculated. tRNAs were clustered accordingly to their relative abundance in the cytoplasm. The tRNA colour code is the same for nucleus and cytoplasm. See also Tables S1 and S2.

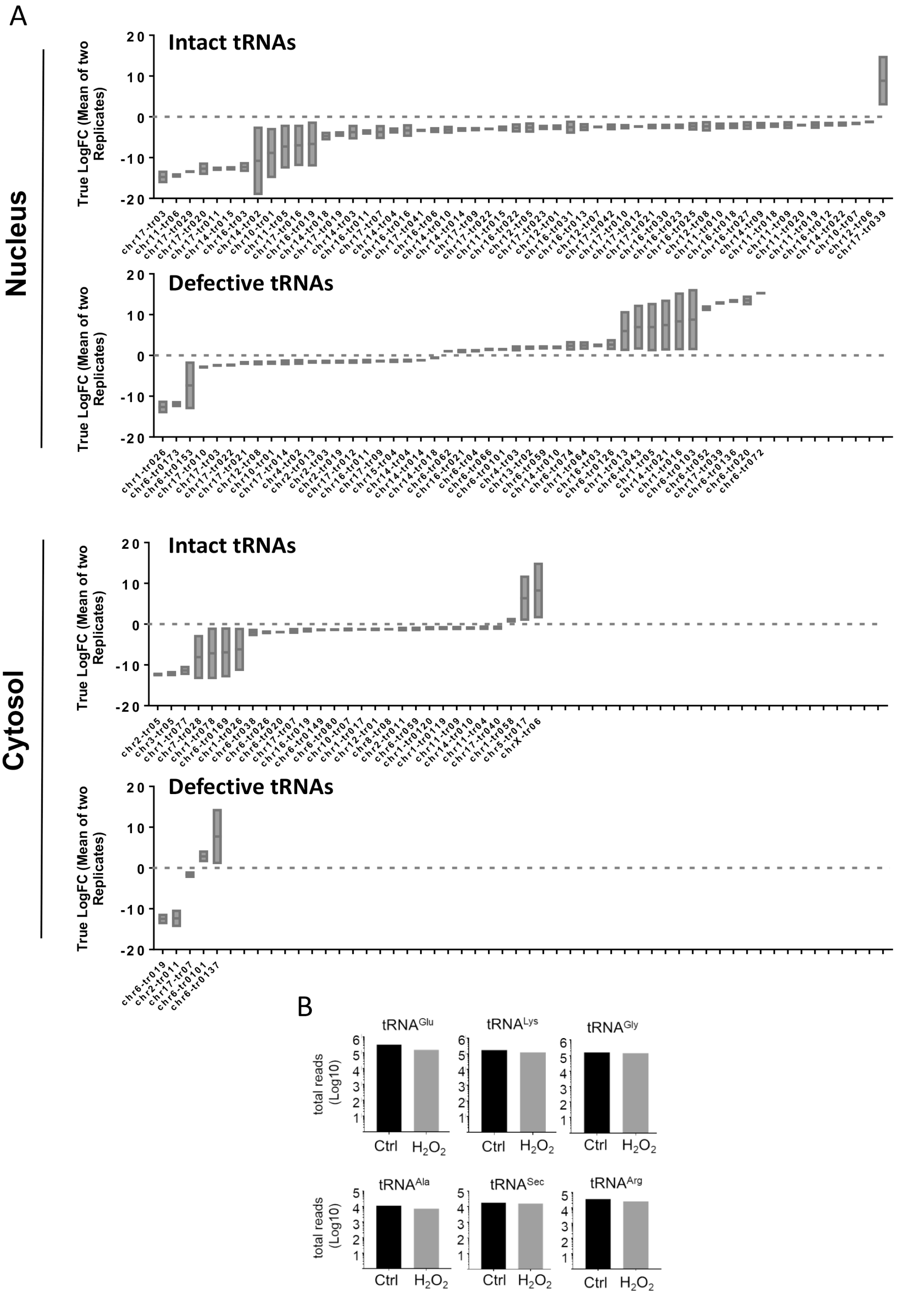

Figure S7 (related to Figure 5 and Table S4). Nuclear and cytosolic distribution of intact and 3’ end truncated

tRNA genes that show a consistent (up or down) LogFC >0.75 in each replicate (True LogFC) upon treatment with 5 mM H_2O_2 for 2 hours in the presence of ActD.

(A) tRNA-specific reads were mapped to the 3' end of the tRNAs and sorted for intact tRNAs (tRNAs with a mature 3' CCA end) and defective tRNAs (tRNA with an incomplete 3'-end). The data were plotted as average of the two replicates with min/max value. Each bar represents one tRNA gene. (see also Table S4). (B) total tRNA read counts in the nucleus or cytoplasm indicate lack of significant tRNA degradation in H_2O_2-treated relative to control cells.

Table S1

Stress	Stressor	Concentration	Time	HeLa	Neo-NHDF	CD3-T cells
**Oxidative stress**	H_2O_2	0.15, 0.3, 0.6, 1.25, 2.5, 5 (mM)	0.5, 1, 2, 3, 4 (hours)	+++	++	++
**Genotoxic stress**	Methylmethane Sulfonate (MMS)	5 mM	15, 30, 60, 120 (minutes)	+	-	++
**Translation inhibition**	Puromycin	3 mM	5, 7.5, 10, 15, 30 (minutes)	++	-	++
**Metabolic stress**	Glucose deprivation		1, 2, 3, 6, 12, 24, 48 (hours)	-	-	-
**Viral infection**	IFNα	1.15 x 10^4 U/μL	1, 2, 3, 6, 12, 24, 48 (hours)	-	-	-
**Inflammation**	TNFα	10 ng/mL	1, 2, 3, 4 (hours)	-	-	-
**Heat shock**	42 ^{0}C		15, 30, 60 (minutes)	-	ND	ND
**Low pH**	NaOH	0.1% (1M)	1, 2, 3 (hours)	-	ND	ND
**High pH**	HCl	0.1% (1M)	1, 2, 3 (hours)	-	ND	ND
**Osmotic pressure**	Sucrose	300 mM	1, 2, 3 (hours)	-	ND	ND
