# Supplementary material for: Oxidative Stress Triggers Selective tRNA Retrograde Transport in Human Cells during the Integrated Stress Response
Source: Cell Rep. 2019 Mar 19;26(12):3416–3428.e5. doi: 10.1016/j.celrep.2019.02.077 (PMC6426654; doi:10.1016/j.celrep.2019.02.077)
Supplement: Document S2. Article plus Supplemental Information [file mmc8.pdf]

Cell Reports

Article

Oxidative Stress Triggers Selective tRNA Retrograde Transport in Human Cells during the Integrated Stress Response

Graphical Abstract

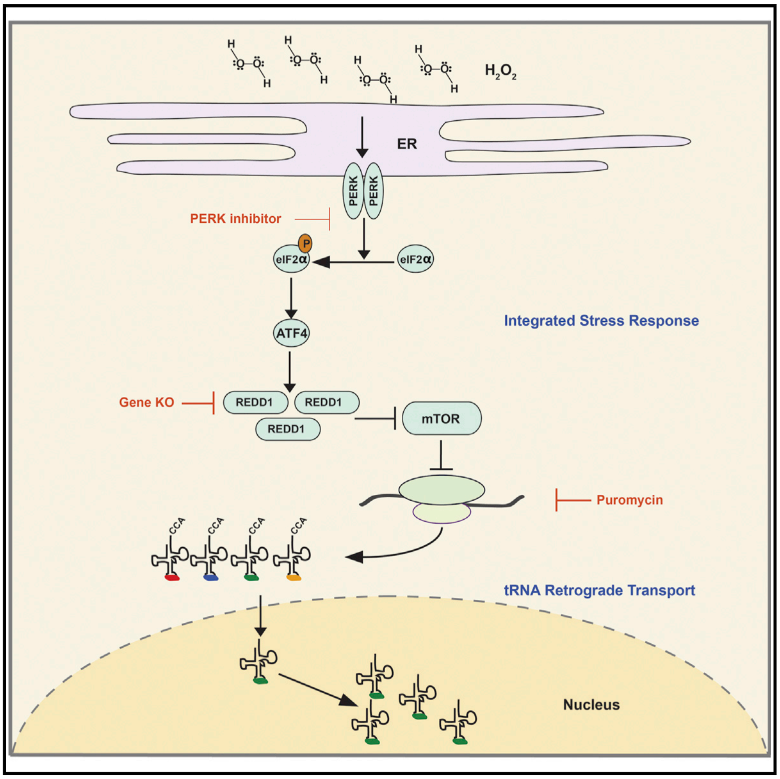

Highlights

- Oxidative stress triggers nuclear import of cytoplasmic tRNAs
- Import is selective for certain tRNAs
- Import requires activation of the unfolded protein response and inhibition of mTOR via REDD1
- tRNA nuclear import is a component of the integrated stress response

Authors

Hagen Schwenzer, Frank Jühling, Alexander Chu, Laura J. Pallett, Thomas F. Baumert, Mala Maini, Ariberto Fassati

Correspondence

a.fassati@ucl.ac.uk

In Brief

Schwenzer et. al discovered that oxidative stress induces nuclear import of cytoplasmic tRNAs. The authors found that this pathway is activated during the integrated stress response. tRNA nuclear import was selective to certain tRNA species and may contribute to the changes in protein translation known to protect cells from stress.

Schwenzer et al., 2019, Cell Reports *26*, 3416–3428
March 19, 2019 © 2019 The Author(s).
https://doi.org/10.1016/j.celrep.2019.02.077

Cell Reports
Article

Oxidative Stress Triggers Selective tRNA Retrograde Transport in Human Cells during the Integrated Stress Response

Hagen Schwenzer,[1,4] Frank Jühling,[2] Alexander Chu,[1] Laura J. Pallett,[1] Thomas F. Baumert,[2,3] Mala Maini,[1] and Ariberto Fassati[1,5,*]
[1]Division of Infection and Immunity, University College London (UCL), London WC1E 6BT, UK
[2]INSERM, U1110, Institut de Recherche sur les Maladies Virales et Hépatiques, 2 Université de Strasbourg, 67000 Strasbourg, France
[3]Nouvel Hôpital Civil, Institut Hospitalo-Universitaire, 67000 Strasbourg, France
[4]Present address: Department of Oncology, University of Oxford, Old Road Campus Research Building, Roosevelt Drive, Oxford OX3 7DQ, UK
[5]Lead Contact
*Correspondence: a.fassati@ucl.ac.uk
https://doi.org/10.1016/j.celrep.2019.02.077

SUMMARY

In eukaryotes, tRNAs are transcribed in the nucleus and exported to the cytosol, where they deliver amino acids to ribosomes for protein translation. This nuclear-cytoplasmic movement was believed to be unidirectional. However, active shuttling of tRNAs, named tRNA retrograde transport, between the cytosol and nucleus has been discovered. This pathway is conserved in eukaryotes, suggesting a fundamental function; however, little is known about its role in human cells. Here we report that, in human cells, oxidative stress triggers tRNA retrograde transport, which is rapid, reversible, and selective for certain tRNA species, mostly with shorter 3′ ends. Retrograde transport of tRNA^{SeC}, which promotes translation of selenoproteins required to maintain homeostatic redox levels in cells, is highly efficient. tRNA retrograde transport is regulated by the integrated stress response pathway via the *PERK-REDD1-mTOR* axis. Thus, we propose that tRNA retrograde transport is part of the cellular response to oxidative stress.

INTRODUCTION

As adaptor molecules for the translational machinery, tRNAs transport their cognate amino acids to cytoplasmic ribosomal complexes, translating the genetic information of mRNA into nascent polypeptide chains (Söll and RajBhandary, 1995). In eukaryotic cells, tRNAs are transcribed by RNA polymerase III within the nucleus. tRNA transcripts undergo a series of post-transcriptional processing steps that are required to yield fully mature and functional tRNAs (Hopper, 2013). As a critical post-transcriptional maturation step, the enzyme tRNA nucleotidyl transferase catalyzes the addition of the ubiquitous CCA nucleotides to the 3′ end of tRNA molecules prior to their export from the nucleus (Wellner et al., 2018).

The dogma of unidirectional movement held that tRNAs are produced inside the nucleus and exported into the cytoplasm to function in protein translation (Söll and RajBhandary, 1995). This tenet of unidirectional transport was initially challenged by the observation that, in yeast, tRNAs are spliced on the surface of mitochondria, but spliced tRNAs were detected inside the nucleus (Yoshihisa et al., 2003). This led to the provocative hypothesis that tRNAs might be exported from the nucleus to the cytoplasm, spliced on mitochondria, and then re-imported into the nucleus, which was later confirmed (Shaheen and Hopper, 2005; Takano et al., 2005). Independently, we were investigating cellular factors driving HIV-1 nuclear import. Using biochemical fractionation approaches, we isolated a fraction able to support HIV-1 nuclear import into human cells *in vitro*. Purified to near homogeneity, the fraction contained tRNAs mostly with defective 3′ CCA ends, and we demonstrated that such defective tRNAs were efficiently imported into the nucleus in an energy-dependent manner (Zaitseva et al., 2006), suggesting that HIV-1 co-opts the physiological import of certain tRNA species for its own nuclear import. tRNA nuclear import has since been reported in rat hepatoma cells (Shaheen et al., 2007), Chinese hamster ovary cells (Barhoom et al., 2011), and human 293T cells (Miyagawa et al., 2012; Watanabe et al., 2013). This pathway is now called ‘‘tRNA retrograde transport’’ (Hopper, 2013).

In S. *cerevisiae*, tRNA retrograde transport is constitutive, whereas re-export of imported tRNAs is regulated by nutrient availability (Chafe et al., 2011; Huang and Hopper, 2014; Murthi et al., 2009). Therefore, this pathway is likely to regulate protein translation by modulating the pool of tRNAs available in the cytoplasm in response to nutrients (Chu and Hopper, 2013). tRNA retrograde transport has also been shown to be a quality control mechanism for tRNA modification and for defective or immature tRNAs (Kramer and Hopper, 2013; Ohira and Suzuki, 2011).

Constitutive tRNA import in S. *cerevisiae* agrees with the notion that, in yeast, tRNA splicing takes place in the cytoplasm and that re-import into the nucleus of spliced tRNAs is required for certain modifications (Ohira and Suzuki, 2011). In human cells, however, tRNA splicing and maturation take place only inside the nucleus (Paushkin et al., 2004; Söll and RajBhandary, 1995). Furthermore, digitonin-permeabilized human cells appear

 © 2019 The Author(s).
This is an open access article under the CC BY license (http://creativecommons.org/licenses/by/4.0/).

to preferentially import *in-vitro*-synthesized tRNAs lacking a complete 3′ CCA end, whereas intact yeast cells import both mature and 3′-end-truncated endogenous tRNAs with similar efficiency (Takano et al., 2005; Zaitseva et al., 2006). Therefore, important differences in how tRNA retrograde transport is regulated in human and yeast cells may exist, which would illuminate new aspects of tRNA biology.

In the present study, we systematically investigated the regulation of retrograde tRNA transport in different human cells. We used a combination of specific tRNA fluorescent *in situ* hybridization (tFISH) to quantitatively characterize tRNA retrograde transport and next-generation sequencing to analyze the global movement of tRNAs. Our results identify tRNA retrograde transport as a component of the cellular defense mechanism against oxidative stress (Spriggs et al., 2010).

RESULTS

Oxidative Stress Induces tRNA Nuclear Accumulation in Human Cells

To investigate the regulation of tRNA retrograde transport in human cells, we exposed HeLa, normal human dermal fibroblasts from neonatal foreskin (neo-NHDF), and primary unstimulated $CD3^+$ T cells to a variety of conditions known to induce stress (Figure 1). We monitored tRNA subcellular localization by tFISH (Shaheen and Hopper, 2005; Shaheen et al., 2007; Takano et al., 2005) using a digoxigenin-labeled oligonucleotide complementary to human $tRNA^{Lys}$, an abundant species tested previously in the same assay (Shaheen et al., 2007). An oligonucleotide probe complementary to $tRNA^{Lys}$ from the bacteria *A. laidlawii* and another oligonucleotide specific for the U5 small nuclear RNA (U5 snRNA) (Gerbi et al., 2003) were used as controls. Under physiological conditions, $tRNA^{Lys}$ was mainly detected in the cytoplasm, whereas U5 snRNA was detected in the nucleus only. The background fluorescence signal was detected with a probe against $tRNA^{Lys}$ from *A. laidlawii* (data not shown).

Each condition was tested using different incubation times and different concentrations of the stressors (Table S1). Oxidative stress is known to affect tRNA modification, expression, and cleavage (Huang and Hopper, 2016) and to repress global protein translation (Spriggs et al., 2010). Therefore, we tested the effect of hydrogen peroxide (H_2O_2), a reactive oxygen species that perturbs protein folding and activates the endoplasmic reticulum (ER) unfolded protein response (UPR) (Harding et al., 2003; Schieber and Chandel, 2014). A hallmark of the UPR is phosphorylation at serine 51 of the α subunit of eukaryotic translation initiation factor 2 (eIF2α), which represses global translational initiation (Harding et al., 2000). We confirmed that H_2O_2 induced eIF2α phosphorylation under our experimental conditions (Figure S1A). When tRNA localization was examined by tFISH, we observed a marked increase in nuclear fluorescence intensity in samples treated with H_2O_2. This effect was robust across the three cell types (Figure 1). Treatment with methylmethane sulfonate (MMS), a nucleic acid-alkylating agent that causes DNA damage and perturbs tRNA modification (Begley et al., 2007; Chan et al., 2010), induced tRNA accumulation in the nucleus of HeLa and primary human T cells but not into neo-NHDF cells (Figure 1). Premature termination of protein synthesis with puromycin has been reported previously to induce nuclear accumulation of *in-vitro*-synthesized tRNAs upon transfection in human 293T cells (Barhoom et al., 2011). We found that puromycin induced accumulation of endogenous $tRNA^{Lys}$ within the nucleus of HeLa and T cells, but this was not detectable in neo-NHDF cells (Figure 1; see also Figure S2 for quantification).

Glucose deprivation is known to induce retrograde tRNA accumulation in S. *cerevisiae* (Hurto et al., 2007; Shaheen and Hopper, 2005; Shaheen et al., 2007; Takano et al., 2015; Whitney et al., 2007); hence, we incubated human cells in the absence of glucose. Glucose deprivation is known to result in de-phosphorylation of 4E-BP1 and reduced protein translation (Sengupta et al., 2010); thus, we examined phosphorylation of 4E-BP1 in our experimental conditions. Although we could confirm the loss of phosphorylated 4E-BP1 (Figure S1B), we could not detect accumulation of $tRNA^{Lys}$ within the nucleus after glucose starvation (Figure 1; Table S1).

Type I interferon (IFN) inhibits protein synthesis via the protein kinase R (PKR) pathway (Sadler and Williams, 2008), and tumor necrosis factor alpha (TNF-α) induces broad changes in protein translation that are linked to ER stress, although the mechanisms are poorly defined (Wang and Kaufman, 2014; Zhai et al., 2008). Therefore, we treated cells with recombinant IFNα or TNF-α. qRT-PCR showed upregulation of the IFN-stimulated gene *IFIT-2* (Levy et al., 1986) and the TNF-α-induced *IL-6* and *I-CAM* genes (Akira et al., 1990) (Figures S1C and S1D), but tFISH did not show nuclear accumulation of $tRNA^{Lys}$ (Figure 1). We also tested osmotic shock and low or high pH but did not detect nuclear tRNA accumulation (Table S1). No $tRNA^{Lys}$ nuclear accumulation was detected after heat shock (Table S1), which is consistent with the observation that this kind of stress only affects $tRNA^{Met}$ (Watanabe et al., 2013). Based on these results, we concluded that oxidative stress was the most consistent inducer of tRNA nuclear accumulation in human cells tested in our experimental settings. Genotoxic stress and translational inhibition induced a cell type-specific response, whereas other stress conditions did not seem to induce significant tRNA nuclear accumulation, at least under the conditions tested (Table S1).

Next we examined the kinetics of tRNA nuclear accumulation after cells were exposed to H_2O_2, MMS, or puromycin. The tFISH signal in the nucleus and cytosol was quantified, and the nuclear:cytosolic fluorescence ratio (N/C) was calculated at each different time point (Figure S2). Accurate quantification of the signal in primary T cells was not possible because of the small size of their cytoplasm. In HeLa cells, tRNA nuclear accumulation was apparent 1 h after exposure to H_2O_2, reaching a peak at 2 h (Figure S2). Similar results were obtained in neo-NHDF and primary T cells (Figures S2 and S3A). A similar response was detected in HeLa cells treated with MMS (Figure S2). Puromycin has been shown previously to change the intracellular distribution of exogenous transfected tRNAs within 30 min (Barhoom et al., 2011); hence, we performed a shorter time course by tFISH, which showed that this drug indeed triggered a faster response than H_2O_2 in HeLa cells, reaching a peak at 7.5 min (Figure S2).

Dose responses were obtained by titrating H_2O_2 and quantifying the tFISH signal as above. Maximal efficacy was reached at 5 mM in HeLa cells, 2.5 mM in neo-NHDF cells (Figure 2), and 0.3 mM in primary T cells (Figure S3B). Thus, although the

OPEN
ACCESS
CellPress

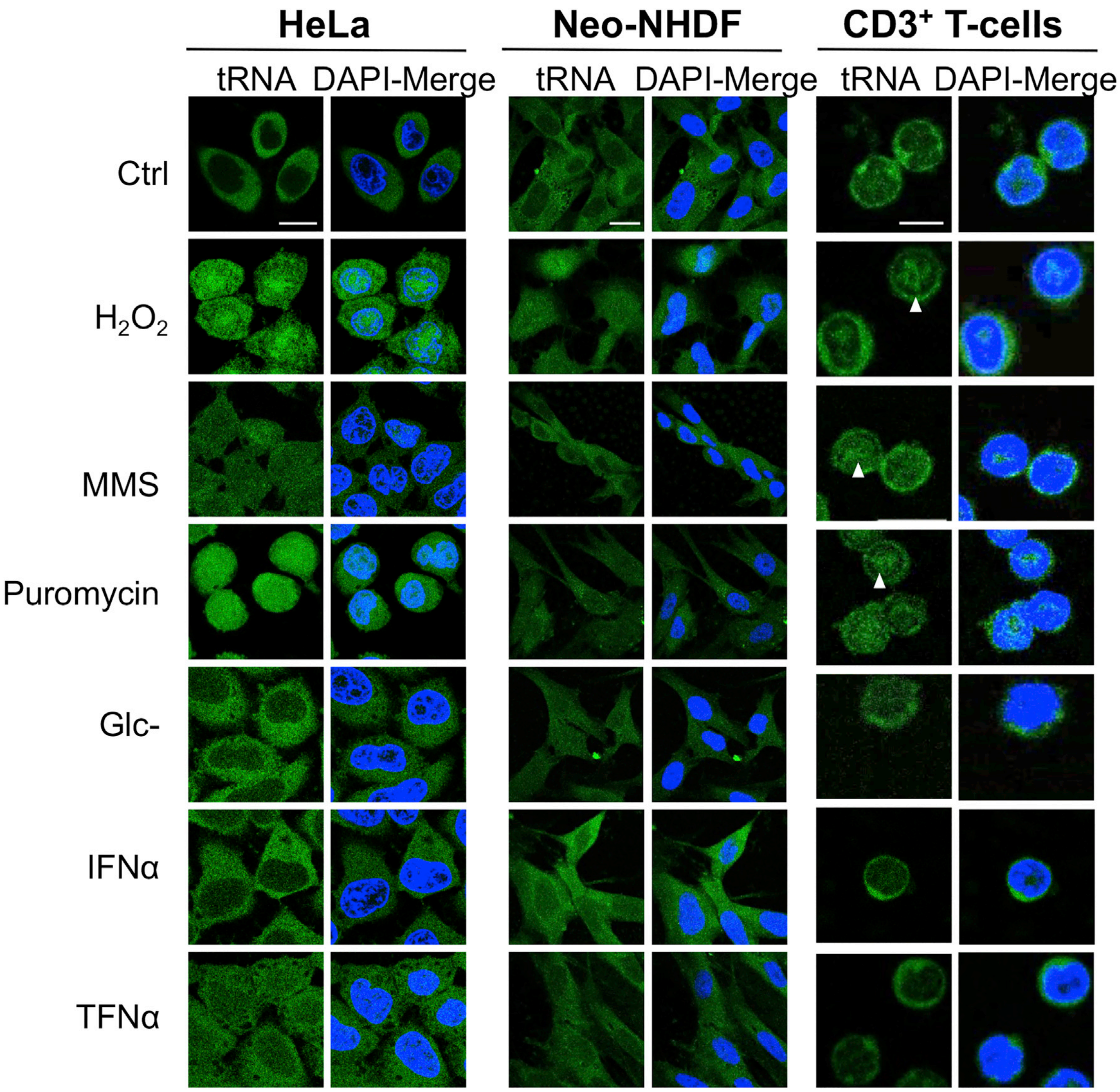

Figure 1. Oxidative Stress, MMS, and Puromycin Induce tRNA Retrograde Transport in Human Cells

A tFISH assay was carried out with a probe specific for $tRNA^{Lys}$ to survey conditions that induce tRNA retrograde transport. Each condition was tested at different time points and with different concentrations of the specified compound. Shown here are H_2O_2 (5 mM for 2 h); MMS (10 mM for 2 h); puromycin (3 mM for 8 min); Glc−, glucose deprivation (24 h); IFNα (1.25 × 10^4 U/mL for 24 h); and TNF-α (1 ng/mL for 2 h). Ctrl, no treatment. Scale bars, 20 μm for HeLa and Neo-NHDF images and 10 μm for CD3+ T cells. Arrowheads point to the intranuclear signal. Representative images of at least three independent experiments are shown. See also Figure S1 and Table S1.

response to H_2O_2 is consistent across different cell types, its threshold is cell-type-dependent, consistent with the notion that cancer cells have a greater tolerance for oxidative stress than normal cells (Wang and Kaufman, 2014).

tRNA Nuclear Accumulation Requires Import of Existing tRNAs and Is Reversible

tRNA nuclear accumulation triggered by oxidative stress might depend on the nuclear import of pre-existing cytoplasmic tRNAs or on a nuclear export block of newly synthesized tRNAs. To distinguish between these two possibilities, we pre-treated cells for 2 h with actinomycin D (ActD) before adding H_2O_2 and then examined the samples by tFISH. ActD is a potent and global inhibitor of *de novo* RNA synthesis, including tRNAs (Shaheen et al., 2007). Hence, if tRNA nuclear accumulation was mainly due to an export block of newly synthesized tRNAs, then one should expect a lower nuclear signal upon treatment with ActD. Conversely, if tRNA nuclear accumulation was mainly due to import of pre-existing cytoplasmic tRNAs, then one should expect no difference. In cells pre-treated with ActD, the level of tRNA nuclear accumulation was either unaffected or higher than in untreated cells (Figure 3A). Furthermore, ActD reduced the cytoplasmic signal in a time-dependent way so that the N/C ratio was higher in ActD-treated than in non-ActD-treated cells (Figures

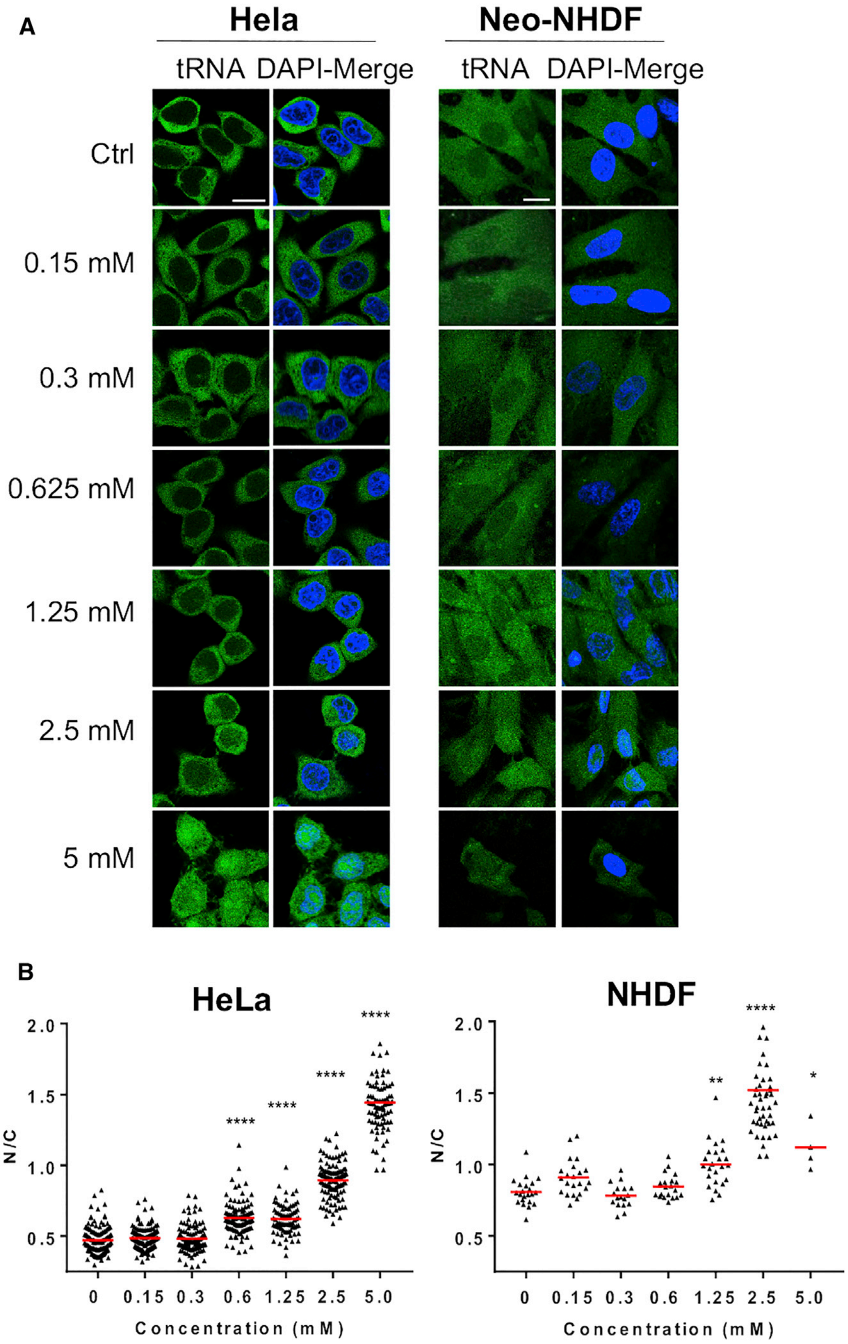

Figure 2. The Effect of H_2O_2 Is Concentration-Dependent

(A) Representative confocal microscopy images showing the changes in nuclear tFISH signal upon addition of H_2O_2 at the indicated concentrations. Scale bar, 20 μm.

(B) ImageJ software was used to quantify the fluorescent signal in the nucleus (N) and cytoplasm (C) and calculate the N/C ratio. Cells were counted from at least 5 randomly chosen images. Each dot corresponds to one cell. Red lines indicate the mean value. Graphs show data from one representative experiment of at least two independent experiments. One-way ANOVA (Dunnett's multiple comparisons test) was used to calculate statistical significance between different groups. *p = 0.05, **p < 0.05, ****p < 0.0001.

See also Figures S2 and S3.

3A and 3B). This suggested that tRNAs were lost from the cytoplasm, presumably because they were imported into the nucleus without being replaced by the export of newly synthesized tRNAs in ActD-treated samples. These results demonstrated that the tFISH signal originates from nuclear import of pre-existing tRNAs. Repression of *de novo* RNA synthesis by ActD was confirmed by qRT-PCR to measure the mRNA levels of *PDX*, *RANBP1*, *TNPO1*, and *MAT1* relative to glyceraldehyde 3-phosphate dehydrogenase (*GAPDH*), a transcript shown previously to be stable under conditions of oxidative stress (Kuwano et al., 2009; Figure 3C).

Of note, co-staining of the ER in cells treated with H_2O_2 and ActD revealed loss of the tFISH signal that co-localized with the ER signal (Figure S4A), suggesting that tRNAs were preferentially imported from the ER into the nucleus.

To determine whether nuclear accumulation of tRNAs induced by oxidative stress is reversible, HeLa cells were treated for 2 h with H_2O_2; then, H_2O_2 was removed, and cells were analyzed by tFISH at different time points (Figures S4B–S4D). We found that removal of H_2O_2 resulted in rapid loss of the nuclear signal, which returned to control levels between 10 and 30 min. Hence, oxidative stress induces fully reversible nuclear accumulation of tRNAs. These results also demonstrate that treatment with H_2O_2 did not cause any irreversible damage to the cells, which maintained their ability to export or degrade nuclear tRNAs following recovery from stress. Indeed, the vast majority of cells treated with H_2O_2 for 2 h were alive (Figure S4E).

Retrograde tRNA Transport Induced by Oxidative Stress Is Selective

Next we sought to understand whether oxidative stress induced retrograde transport of all tRNAs or of specific subsets. To obtain a global view of tRNA retrograde transport, we performed strand-specific deep RNA sequencing using thermostable group II intron reverse transcriptase (TGIRT), a recently developed method that allows high-throughput sequencing of small structured RNAs, including tRNAs (Nottingham et al., 2016; Qin et al., 2016). HeLa cells were treated for 2 h with H_2O_2 in the presence of ActD, harvested, and fractionated into nuclear and cytosolic fractions. The quality of fractionation was confirmed by western blot using antibodies specific for *GAPDH*, protein

OPEN
ACCESS
CellPress

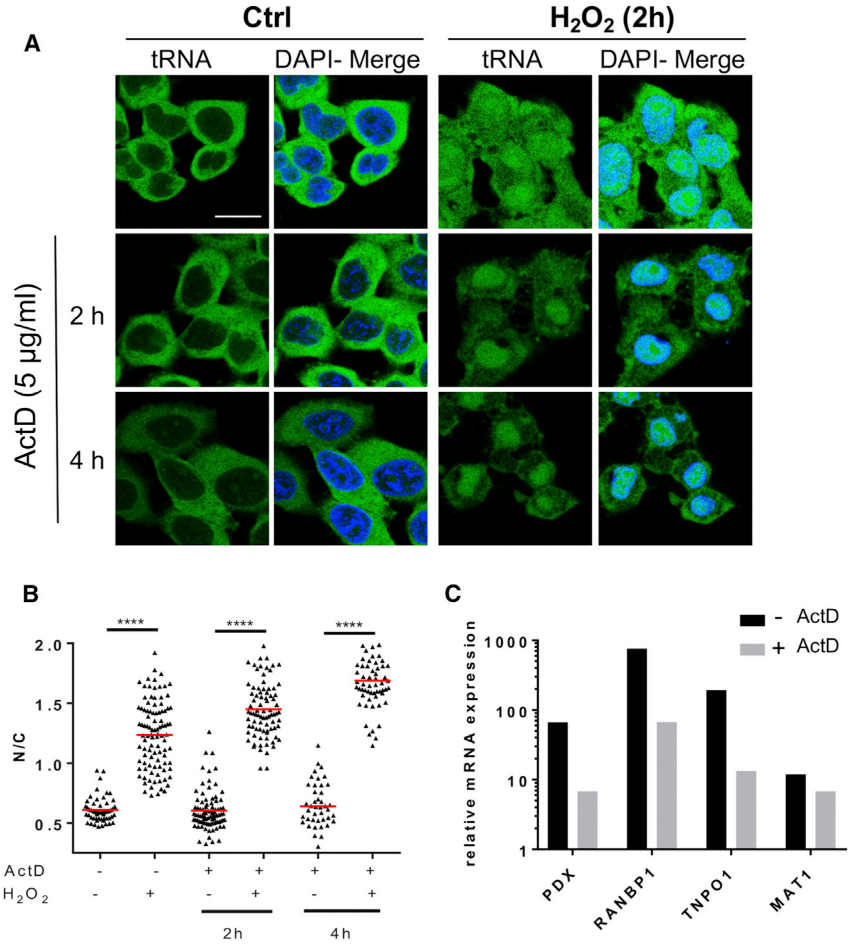

Figure 3. Pre-existing tRNAs Are Imported into the Nucleus upon Exposure to H_2O_2

(A) ActD was added to the cells at the same time as H_2O_2 (5 mM) (2 h total incubation time with ActD) or 2 h before addition of H_2O_2 (4 h total incubation time with ActD). Two h after exposure to H_2O_2, samples were analyzed by tFISH and confocal microscopy to detect $tRNA^{Lys}$. Scale bars, 20 μm.

(B) The N/C ratio was calculated as described in the legend for Figure 1. One-way ANOVA (Dunnett's multiple comparisons test) was used to calculate statistical significance; ****$p < 0.0001$.

(C) Inhibition of *de novo* RNA synthesis under these experimental conditions was confirmed by qRT-PCR to measure the mRNA levels of the indicated genes. Expression levels are relative to *GAPDH* mRNA levels.

See also Figure S4.

disulfide isomerase (PDI), and lamin-B1 (lamin) as markers for the cytosol, the ER, and the nucleus, respectively (Figure 4A). Between 6 and 12 million reads were obtained for each sample. Total reads were mapped to the ENSEMBLE non-coding RNA (ncRNA) set that includes more than 300 unique tRNA gene sequences obtained from the Genomic tRNA Database (gtRNAdb) (Lowe and Chan, 2016). Initial optimization for the preparation of the sequencing library revealed that treatment with demethylase reduced the overall yield and tended to degrade the tRNA 3′ end, which was critical for our analyses, presumably because of difficult-to-remove contamination with RNase. Because we did not treat the samples with demethylase, tRNA modifications might have prematurely blocked reverse transcription, introducing mapping artifacts (Clark et al., 2016; Zhou et al., 2018). To examine this issue, we assessed coverage of each single position in a tRNA covariance model using more than 1,000 tRNA alignments from RFAM (http://rfam.xfam.org/) and checked against known RNA modifications (Boccaletto et al., 2018). The overall coverage was good, although it was lower at the 5′ end between nucleotides 1 and 30, presumably because of stalling of the enzyme during the reverse transcription step. This higher rate of "stopping" at the 5′ end has been observed previously (Clark et al., 2016). However, this lower 5′ end coverage did not prevent mapping of the tRNAs (Figures S5 and S6A).

The majority of mapped reads (62%–83%) belonged to small non-coding RNAs, and up to 30% of these could be mapped to tRNAs (Figure S6A). However, only ≅5% of the total reads could be mapped in our third biological replicate; therefore, this replicate was not included in subsequent analyses. Analysis of the ten most abundant reads revealed clear enrichment of nucleus- and nucleolus-specific small ncRNAs in the nuclear fraction and of cytosol-specific small RNAs, including several mitochondrion-encoded tRNAs, in the cytoplasmic fraction, further confirming the high quality of the fractionation (Figure S6B).

To assess whether the overall composition of the tRNA pool within the cytosol and nucleus changed upon treatment with H_2O_2, all normalized unique reads were counted and clustered according to tRNA amino acid usage (Figure 4B; Figure S6C) and isoacceptors (Table S2). Their relative distribution inside the nucleus and the cytoplasm was calculated for each replicate: $tRNA^{Lys}$, $tRNA^{Gly}$, $tRNA^{Glu}$, and $tRNA^{Asp}$ were the most abundant species, covering more than 50% of all tRNA reads. This result matches a recently published study that used TGIRT to quantify tRNA isoacceptor abundance in the Universal Human Reference RNA (Nottingham et al., 2016). In our samples, the least abundant tRNAs were $tRNA^{Phe}$, $tRNA^{Tyr}$, $tRNA^{Cys}$, and $tRNA^{Trp}$. Under oxidative stress conditions, the proportion of tRNAs for selenocysteine, alanine, serine, threonine, and isoleucine in the nucleus was higher relative to glycine and aspartic acid. This effect was observed in each biological replicate (Figure 4B; Table S2). The relative proportion of $tRNA^{Lys}$ increased in the nucleus upon oxidative stress; however, this was observed in one biological replicate only (Figure 4B).

These results suggested that tRNA retrograde transport might be selective for certain tRNA species. Thus, we sought

OPEN
ACCESS
CellPress

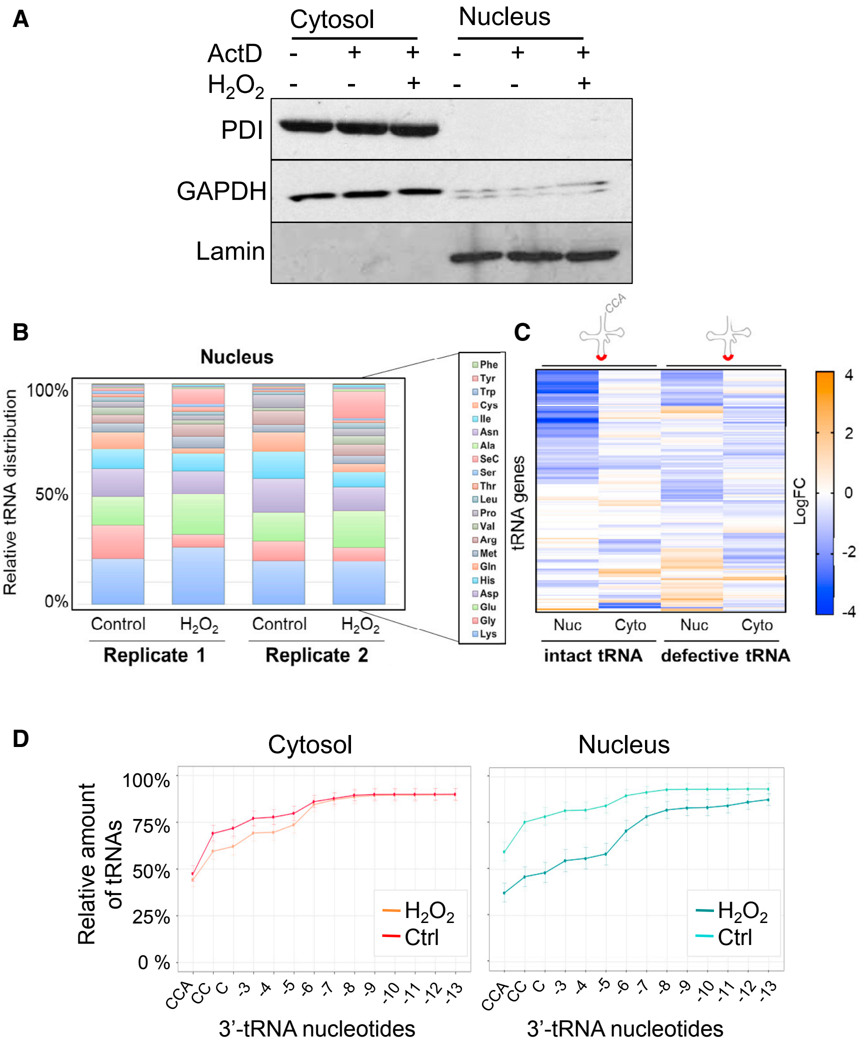

Figure 4. Oxidative Stress Induces Changes in the Overall Composition of the Cytosolic and Nuclear tRNA Pools

HeLa cells were treated with ActD in the presence or absence of 5 mM H_2O_2 for 2 h. Cells were fractionated into a nuclear and cytosolic fraction, RNA extracted from each fraction, and next-generation sequencing (TGIRT) of small RNAs was performed.

(A) Western blot of nuclear and cytosolic fractions with antibodies detecting marker proteins for the nucleus and cytoplasm. *GAPDH*, glyceraldehyde 3-phosphate dehydrogenase; PDI, protein disulfide isomerase; lamin, lamin-B1.

(B) Total RNA reads were mapped to 323 unique tRNA genes. All reads mapping to the same tRNA species were counted and combined, and their relative distribution was calculated. tRNAs were clustered according to their relative abundance in the nucleus.

(C) Heatmap showing the Log_2 fold change (Log_2FC) of tRNA-specific reads in nuclear or cytoplasmic fractions of cells treated with H_2O_2 relative to untreated cells. tRNA-specific reads were mapped to the 3′ end of the tRNAs and sorted for intact tRNAs (with a full 3′ CCA end) and defective tRNAs (with an incomplete 3′ end). A threshold base mean of more than 20 reads was applied across all samples.

(D) Cumulative abundance of intact or 3′ end-truncated tRNAs in the cytosolic and nuclear fractions upon treatment with H_2O_2. Sequencing reads were mapped to the 3′ end of the tRNAs at single-nucleotide resolution for the first 13 nucleotides. The relative amount of each tRNA species is shown for each missing nucleotide at the 3′ end. See also Figures S5 and S6 and Tables S2, S3, S4, and S5.

to address the question of selectivity. Furthermore, we previously reported that, in digitonin-permeabilized human cells, *in-vitro*-synthesized tRNAs lacking a complete 3′ CCA end were preferentially imported into the nucleus relative to tRNAs with a full 3′ CCA end (Zaitseva et al., 2006). However, it was not known whether this applies to endogenous tRNAs as well. Thus, we sought to understand the type of tRNAs (intact or truncated at the 3′ end) that undergo retrograde transport under oxidative stress.

To this end, we examined the distribution of each intact (tRNA with a mature 3′ CCA end) and defective (tRNAs missing at least one nucleotide at their 3′ end) gene in the nucleus and cytoplasm upon treatment with H_2O_2. We mapped all reads to intact and 3′-truncated tRNA transcript versions and calculated the Log_2 fold change (Log_2FC). For greater stringency, we counted only tRNA genes with a sequencing depth of at least 20 reads in the cytosolic control samples, which resulted in a pool of 208 unique tRNA genes suitable for further analyses (Table S3). This analysis revealed a cluster of truncated tRNAs enriched and another cluster of intact tRNAs reduced in the nuclear fractions (Figure 4C; Table S4), with 58 tRNA genes reaching statistical significance ($p < 0.05$) and 19 tRNA genes reaching an adjusted p value (adjp) of less than 0.05 (Table S3). Changes in the cytosol were modest, and no tRNA gene reached statistical significance (Figure 4C; Table S3). To further improve confidence in the results, we focused on tRNAs that showed a consistent positive or negative Log_2FC >0.75 in both biological replicates. Using this cutoff, in the nuclear fraction we found one intact tRNA ($tRNA^{Ala}$) and 24 defective tRNAs, including $tRNA^{Sec}$, $tRNA^{Ala}$, $tRNA^{Val}$, $tRNA^{Asn}$, $tRNA^{Ser}$, $tRNA^{Lys}$, and $tRNA^{Ile}$, that were increased by oxidative stress, whereas, in the cytosolic fraction, we found two intact ($tRNA^{Ala}$ and $tRNA^{Ile}$) and three defective tRNAs, which were increased (Figure S7; Table S5). $tRNA^{Sec\ (TCA)}$ (cca_chr19-tr09), one of the most highly changed tRNA following treatment with H_2O_2, was reduced by an average of 2.7-fold in the cytoplasm and increased by an average of 8.6-fold in the nucleus (Tables S2 and S4).

To further examine the nature of these defective tRNAs, sequencing reads were mapped to the 3′ end of the tRNAs at single-nucleotide resolution, and the relative distribution of

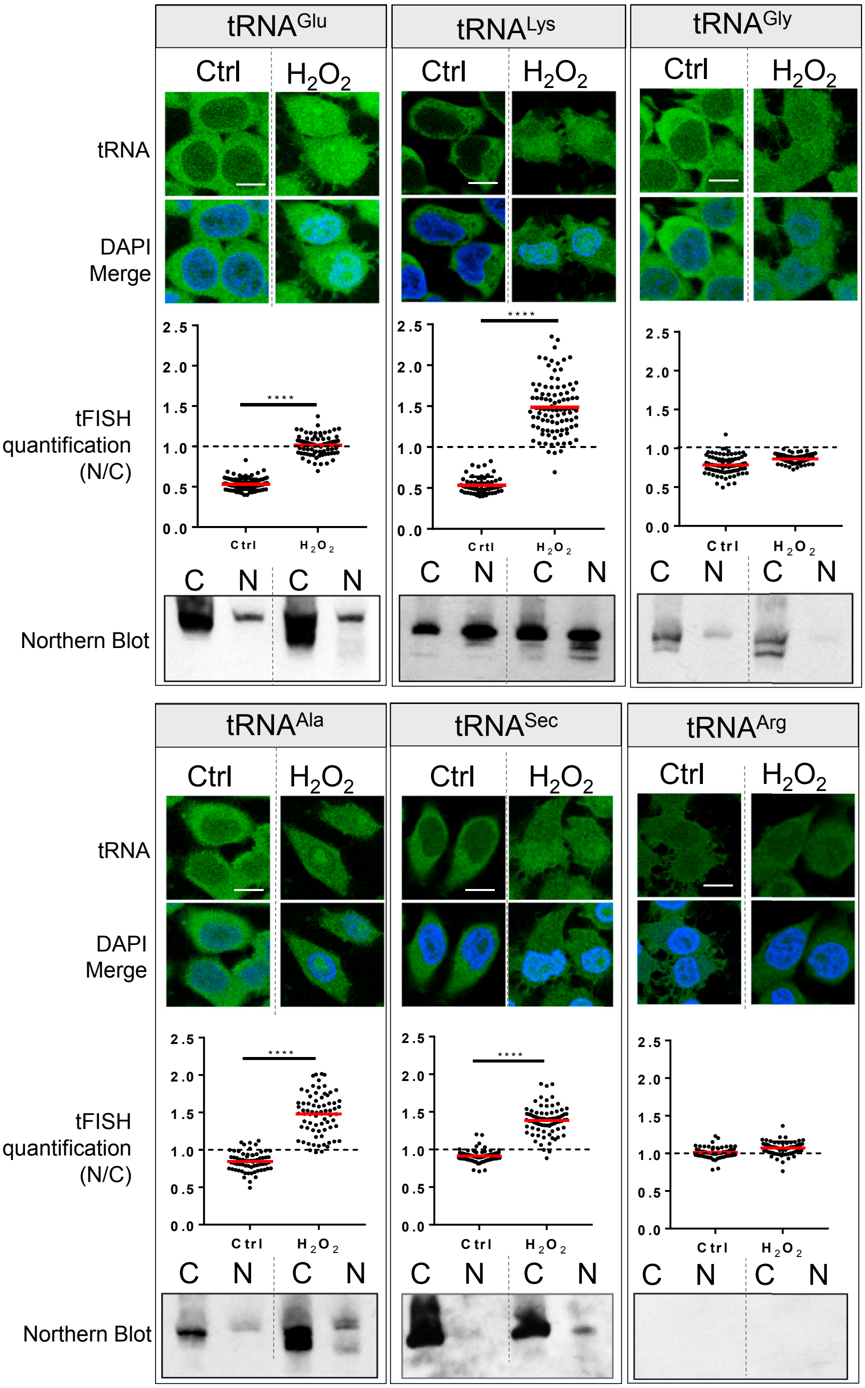

Figure 5. tRNA Retrograde Transport in Human Cells Is Selective

Top: cells were exposed to 5 mM H_2O_2 for 2 h without ActD and analyzed by tFISH with the indicated tRNA probe. Representative confocal microscopy images of the tFISH assay are shown. Scale bars, 10 μm. Center: the N/C ratio was calculated as described in the legend for Figure 1. The graphs show data from one representative experiment of at least two. One-way ANOVA (Dunnett's multiple comparisons test) was used to calculate statistical significance; ****$p < 0.0001$. Bottom: cells were fractionated into nuclear (N) and cytosolic (C) fractions. Total RNA was extracted from each fraction and analyzed by northern blot using the same tRNA-specific probes employed for the tFISH. Each image is representative of at least two independent experiments See also Figure S7.

each tRNA in the nucleus and cytosol was calculated for control and H_2O_2-treated samples (Figure 4D). In the cytosolic control, approximately 50% of reads mapped to intact tRNAs, whereas the remaining portion lacked between 1 to 6 nt at their 3′ end. Oxidative stress had a marginal effect on the proportion of reads mapping to defective tRNAs. In the nucleus, however, only 30% of reads mapped to intact tRNAs in H_2O_2-treated cells compared with 60% of reads in control cells. Many defective tRNAs in the nucleus lacked between 5 and 8 nt at their 3′ end (Figure 4D). Overall, these analyses suggested that nuclear accumulation involved predominantly certain tRNA species with truncated 3′ ends, although it cannot be excluded that some 3′ end shortening also occurred in the nucleus following oxidative stress.

To confirm the selectivity of the tRNA retrograde transport induced by oxidative stress, we examined, by tFISH, four tRNAs that showed robust nuclear accumulation according to RNA sequencing (RNA-seq) (tRNA^{Glu}, tRNA^{Ala}, tRNA^{Lys}, and tRNA^{Sec}) and two that did not (tRNA^{Gly} and tRNA^{Arg}). In agreement with the RNA-seq results, tRNA^{Ala}, tRNA^{Sec}, tRNA^{Glu}, and tRNA^{Lys} showed significant nuclear accumulation whereas tRNA^{Gly} and tRNA^{Arg} did not (Figure 5). This result could not be explained by the relative abundance of the tRNAs because, for example, tRNA^{Gly} was 28 times more abundant than tRNA^{Ala}, but tRNA^{Ala} clearly accumulated into the nuclei and tRNA^{Gly} did not (Figure 5). We also sought to confirm by other means the presence of truncated tRNAs in the nucleus of cells treated with H_2O_2. To this end, cells were treated for 2 h with H_2O_2, fractionated into nuclear and cytoplasmic extracts and examined by northern blot (Figure 5). The results confirmed the presence of bands of smaller size relative to their corresponding intact tRNAs for tRNA^{Glu}, tRNA^{Lys}, tRNA^{Ala}, and, possibly, tRNA^{Sec}, whereas the results were inconclusive for tRNA^{Arg} because of the insufficient sensitivity of the northern blot for this particular tRNA (Figure 5). Importantly, the smaller bands were detected in the nuclear fractions of cells treated with H_2O_2 but not in untreated cells.

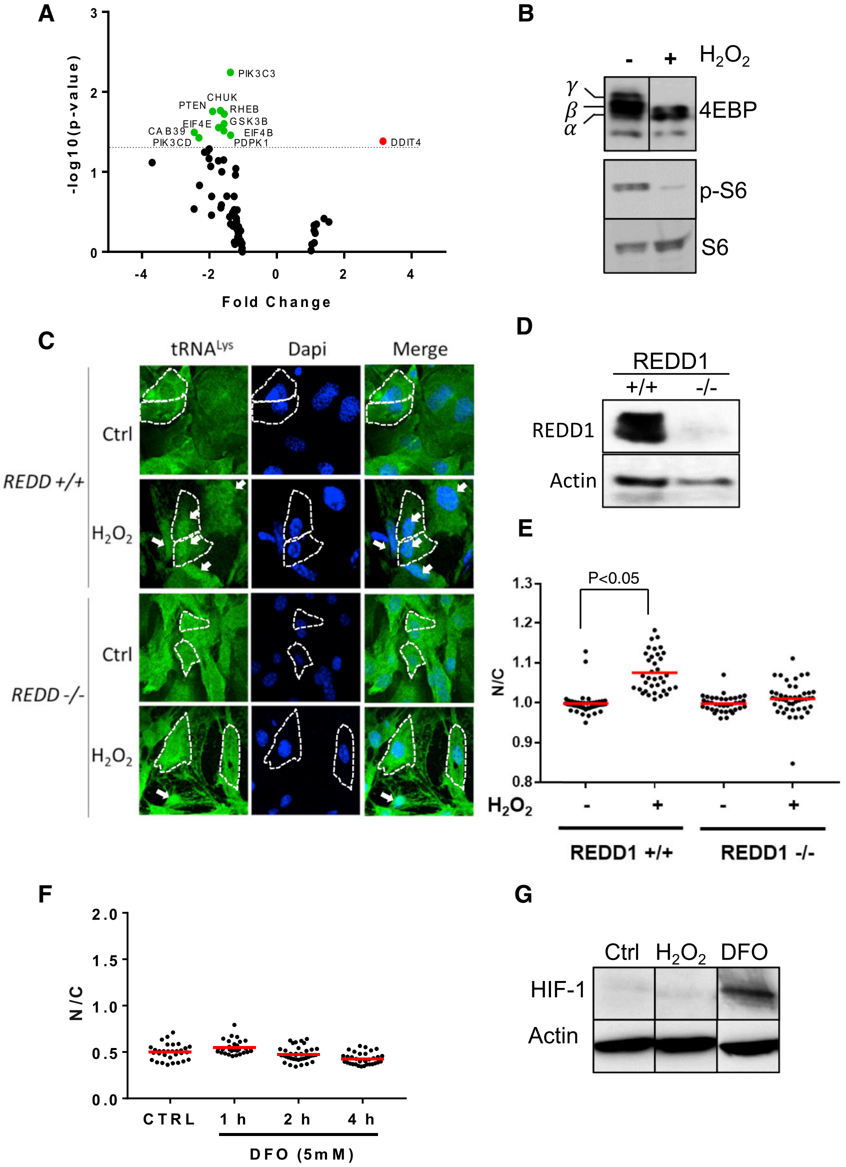

Figure 6. *REDD1/DDIT4* Regulates tRNA Retrograde Transport

(A) HeLa cells were exposed to 5 mM H_2O_2 for 2 h, and changes in RNA expression were analyzed using a pre-coated 96-well qPCR assay with probes specific for the mTOR signaling pathway. Significant up and downregulated genes (FC >1.5, $p \leq 0.05$) are indicated in red and green, respectively.

(B) Inhibition of the mTOR pathway was confirmed by western blot to detect phosphorylation of the translation inhibitor 4EBP and the ribosomal protein S6 (Ser235/236).

(C) *REDD1* knockout MEFs ($REDD^{-/-}$) showing reduced tRNA retrograde transport when treated with 3 mM H_2O_2 compared with wild-type cells ($REDD^{+/+}$). Arrows point to cells with a positive nuclear signal. Scale bar, 10 μm.

(D) *REDD1* protein expression analysis by western Blot for $REDD^{+/+}$ and $REDD^{-/-}$ cells.

(E) The N/C ratio was calculated as described in the legend for Figure 1. Unpaired two-tailed Student's t test was used to calculate statistical significance.

(F) Cells were incubated for 4 h with the hypoxia mimetic agent desferrioxamine (DFO, 5 mM), and tRNA nuclear accumulation was detected by tFISH and quantified by ImageJ. The graphs show data from one representative experiment of three.

(G) Stabilization of HIF-1 was detected by western blot in parallel experiments, and actin was used as a loading control.

See also Table S6.

tRNA Retrograde Transport Is Regulated by *REDD1* and *mTOR*

Oxidative stress affects pathways that ultimately result in inhibition of global protein translation and induction of specific stress response proteins, which protect cells from deleterious damage (Gebauer and Hentze, 2004; Harding et al., 2000, 2003). To better understand the effect of H_2O_2 in our cellular model, we sought to profile the expression of marker genes of mammalian target of rapamycin (mTOR), one of the key pathways that affect protein translation during oxidative stress (Saxton and Sabatini, 2017). Cells were treated for 2 h with H_2O_2, and RNA was extracted and reverse-transcribed. Changes in gene expression were quantified by real-time qPCR (Table S6). Notably, the only significantly upregulated gene in the network was *REDD1* (also called *DDTI4*) (Figure 6A), which senses oxidative stress and inhibits mTOR through the *TSC1* and *TSC2* complex (Brugarolas et al., 2004). Conversely, the most significantly downregulated genes were *PTEN*, *PDK1*, *PIK3C2*, and *PIK3CD* (Figure 6A), which stimulate mTOR via AKT activation. Thus, the transcriptional changes converged to reduce mTOR activity, and this was confirmed by the lower levels of phosphorylated S6 and 4EBP, two key targets of mTOR, in H_2O_2-treated cells relative to control cells (Figure 6B).

REDD1 acts upstream of *mTOR*; it is an important sensor of cellular stress whose expression is rapidly upregulated to promote cell survival (Brugarolas et al., 2004; Dennis et al., 2013). We therefore hypothesized that *REDD1* is essential for tRNA retrograde transport. To test this hypothesis, we obtained wild-type and $REDD1^{-/-}$ mouse embryonic fibroblasts (MEFs) (Brugarolas et al., 2004; Figures 6C and 6D), exposed them to H_2O_2 for 2 h, and performed tRNA FISH. We found a significantly lower nuclear signal ($p < 0.05$) in $REDD1^{-/-}$ MEFs relative to wild-type cells (Figures 6C and 6E), confirming the role of this gene in tRNA retrograde transport. *REDD1* was initially described as a hypoxia-induced target gene of HIF-1 (Shoshani et al., 2002), which prompted us to examine whether HIF-1 was stabilized by H_2O_2 under our experimental conditions and, therefore, could induce *REDD1* expression. However, western blot analysis did not show stabilization of HIF-1 under H_2O_2. Conversely, desferrioxamine, a well-known HIF-1 stabilizer (Woo et al., 2006), induced HIF-1

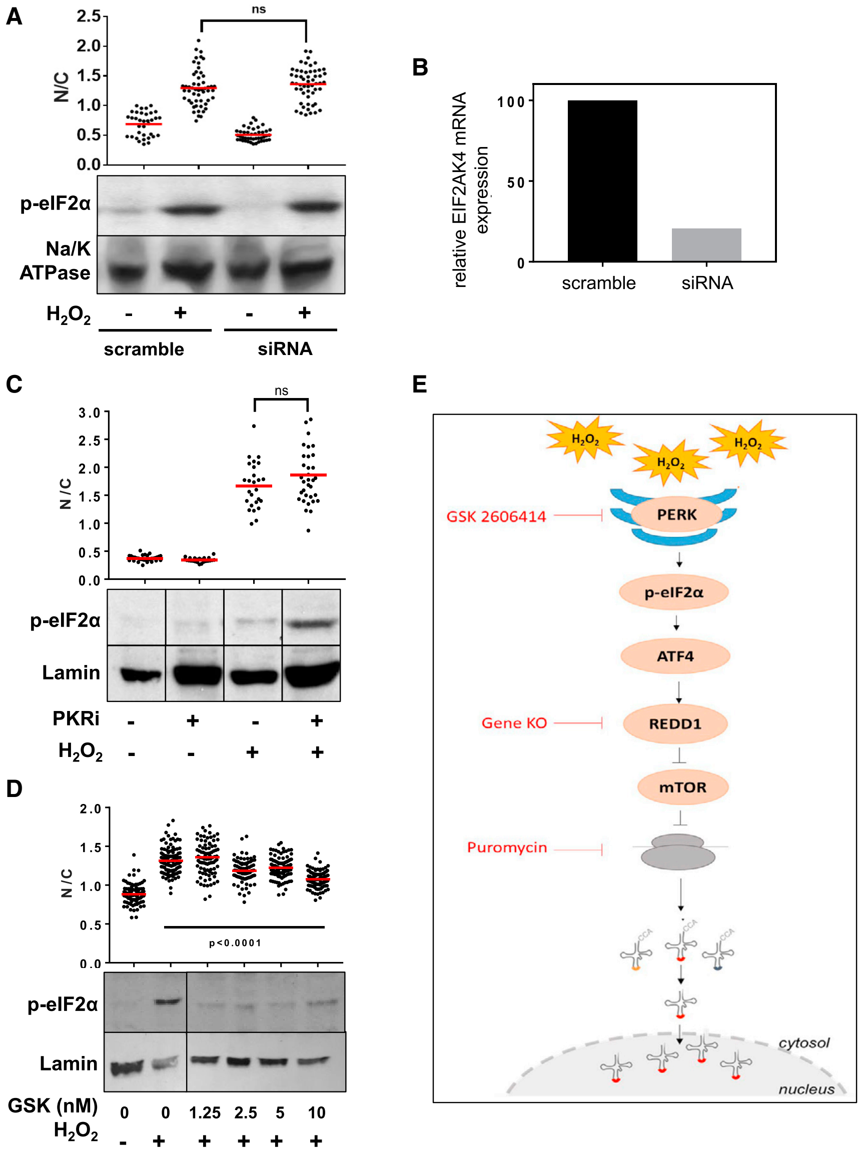

Figure 7. *PERK* Regulates tRNA Retrograde Transport

(A) Top: cells treated with siRNA targeting *GNC2* (*EIF2AK4*) or with scrambled siRNA were incubated with 5 mM H_2O_2 for 2 h, and tRNA nuclear accumulation was detected by tFISH and quantified by ImageJ. Bottom: western blot showing S51 phosphorylation of eIF2α in the presence or absence of H_2O_2 in mock and *GCN2/EIF2AK4*-depleted cells.

(B) qRT-PCR to detect *EIF2AK4* mRNA in cells transfected with the targeting or scrambled siRNAs.

(C) Top: cells were treated with 5 mM H_2O_2 for 2 h in the presence or absence of the imidazole-oxindole PKR inhibitor C16 (PKRi, 300 nM), and tRNA nuclear accumulation was detected by tFISH and quantified by ImageJ. Bottom: S51phosphorylation of eIF2α was detected by western blot, and lamin was used as a loading control.

(D) Top: cells were incubated with 5 mM H_2O_2 for 2 h in the presence of different concentration of the PERK inhibitor GSK 2606414 (GSK), and tRNA nuclear accumulation was detected by tFISH and quantified by ImageJ. Bottom: S51 phosphorylation of eIF2α was detected by western blot, and lamin was used as a loading control.

(E) Schematic summary of the pathways regulating tRNA retrograde transport.

Statistical significance was calculated by unpaired two-tailed Student's t test (A and C) or by one-way ANOVA (Dunnett's multiple comparisons test) (D).

stabilization but did not recapitulate H_2O_2-induced tRNA retrograde transport (Figures 6F and 6G). These results indicate that tRNA retrograde transport is disconnected from HIF-1.

Beside HIF-1, the transcription of *REDD1* is also regulated by *ATF4*, which is a transcription factor induced by the UPR stress signaling pathway (Whitney et al., 2009). A hallmark of the UPR is eIF2α phosphorylation (Harding et al., 2000), and we have already observed that tRNA retrograde transport induced by oxidative stress occurs in parallel with eIF2α phosphorylation (Figure S1). This suggested that activation of the UPR pathway might also regulate tRNA retrograde transport by linking oxidative stress to the upregulation of *REDD1.* To further investigate this potential link, we inhibited each of the three kinases that can independently phosphorylate eIF2α in the UPR pathway: *GNC2* (called *EIF2AK4* in *Homo sapiens*), *PKR*, and *PERK* (Harding et al., 2002). Small interfering RNA (siRNA)-mediated depletion of *GNC2* (Figures 7A and 7B) or a selective small-molecule PKR inhibitor (Jammi et al., 2003; Figure 7C) did not affect eIF2α phosphorylation induced by H_2O_2 or had any effect on tRNA retrograde transport. However, when we treated cells with a selective inhibitor of PERK (Axten et al., 2012), we observed reduced eIF2α phosphorylation and a dose-dependent inhibition of tRNA nuclear accumulation (Figure 7D). Altogether, these results suggest that tRNA retrograde transport induced by oxidative stress depends on crosstalk between *PERK* and *mTOR* that is orchestrated by *REDD1*.

DISCUSSION

Our study shows that tRNA retrograde transport is activated by H_2O_2 in a concentration- and time-dependent manner and that this pathway is selective for certain tRNA species, mainly involves tRNAs with a defective 3′ end, and is connected to the integrated stress response.

Our results based on RNA-seq suggest that tRNA retrograde transport is selective for certain tRNA genes, mainly with a truncated 3′ end. Caution is required when interpreting the RNA-seq results because only a relatively small proportion of tRNAs reached statistical significance. The large number of tRNA genes

analyzed and the few biological replicates it was possible to sequence reduced the overall power of the analysis. Nonetheless, a consistent pattern emerged from the two biological replicates. and the specific nucleo-cytoplasmic distribution of several tRNAs was confirmed by tFISH and northern blotting, suggesting that the tRNA-seq revealed a genuine trend. Currently we do not know the mechanisms of this selectivity; however, we note that, in yeast, several tRNA export factors have been described recently that show remarkable selectivity for certain tRNAs (Chatterjee et al., 2017). Presumably, tRNA retrograde transport in human cells may also depend on several importers, each having a specific substrate.

Our data also indicate that, in human cells, tRNA retrograde transport involves mainly truncated tRNAs lacking a complete 3′ end, which is consistent with our previous findings in digitonin-permeabilized cells (Zaitseva et al., 2006). Although we do not know how these defective tRNAs are generated, in yeast, plant, and mammalian cells, oxidative stress has been shown to induce rapid cleavage of tRNAs to produce tRNA halves (called tiRNAs [tRNA stress-induced fragments]) (Thompson et al., 2008; Yamasaki et al., 2009). Transfection of these tiRNAs repressed global protein translation in an eIF2α phosphorylation-independent way, suggesting that tiRNAs are a component of a separate stress response pathway (Ivanov et al., 2011; Yamasaki et al., 2009). Furthermore, in *Tetrahymena*, small tRNA fragments are found associated with Piwi proteins upon starvation (Couvillion et al., 2010). The endoribonuclease angiogenin generates tiRNAs (Fu et al., 2009; Ivanov et al., 2011; Yamasaki et al., 2009) and cleaves the terminal A residue of the 3′ end CCA tail under oxidative stress (Czech et al., 2013); hence, it may generate at least part of the truncated tRNAs we observed. However, the heterogeneity of the tRNA 3′ ends also suggests that a 3′ to 5′ exonuclease might be involved.

Several conditions triggered tRNA retrograde transport in human cells (namely, puromycin, MMS, and H_2O_2), but only H_2O_2 activated the pathway in all cell types tested, indicating that this response may be universal. Of note, the H_2O_2 concentration required to activate retrograde transport was higher in HeLa cells relative to neo-NHDF and primary T cells, which is consistent with the previously reported lower sensitivity of cancer cells to oxidative stress (Cubillos-Ruiz et al., 2017). In yeast, tRNA nuclear accumulation is observed under conditions of glucose starvation (Chafe et al., 2011; Huang and Hopper, 2014; Whitney et al., 2007). Human cells do not seem to trigger tRNA retrograde transport in response to this particular stress. However, based on our results, we cannot conclude that, in human cells, tRNA retrograde transport is insensitive to nutrient deprivation because we only detected $tRNA^{Lys}$ by tFISH, and a wider survey of different tRNAs in different cell types and nutrient deprivation conditions would be required. Instead, our results point to the intriguing possibility that tRNA retrograde transport might be a cell type-specific response.

H_2O_2 is a reactive oxygen species that mediates intracellular signaling by promoting reversible oxidation of cysteine residues within proteins (Schieber and Chandel, 2014; Winterbourn and Hampton, 2008). When cells are exposed to high concentrations of H_2O_2, cysteine oxidation becomes irreversible, causing permanent protein damage (Schieber and Chandel, 2014). Accumulation of damaged proteins activates the UPR, leading to phosphorylation of eIF2α, which reduces translational initiation and activates the ATF4-dependent transcriptional program (Harding et al., 2000). Our results indicate that tRNA retrograde transport depends on the integrated stress response pathway and involves PERK-mediated phosphorylation of eIF2α and transcriptional upregulation of *REDD1*, leading to mTOR inhibition of S6K and 4EBP (Ait Ghezala et al., 2012; Brugarolas et al., 2004). Of note, reduced phosphorylation of S6K and 4EBP alone was not sufficient to trigger $tRNA^{Lys}$ nuclear accumulation (for example, upon glucose deprivation), suggesting that tRNA retrograde transport requires concomitant UPR activation. Activation of these pathways results in inhibition of global protein translation and concomitant induction of specific stress response proteins, which maintain cellular homeostasis and promote cell survival for a period of time until the specific insult is removed (Kroemer et al., 2010; Pakos-Zebrucka et al., 2016; Spriggs et al., 2010). Therefore, we propose that, in human cells, tRNA retrograde transport may be part of the integrated stress response to protect cells from damage caused by reactive oxygen species. This possibility is consistent with the rapid induction of tRNA nuclear accumulation during treatment with H_2O_2, its rapid reversibility, and its link to the UPR via *PERK* and phosphorylation of eIF2α. This idea provides a framework to explain the selectivity of the pathway because sequestration of specific tRNAs into the nucleus might conceivably contribute to the re-shaping of protein translation that takes place during oxidative stress.

In support of this notion, oxidative stress induced significant nuclear accumulation of both intact and truncated $tRNA^{SeC}$. Seleno-cysteine (SeC) incorporation competes with the translation termination reaction, instead favoring translation of selenoproteins required to maintain homeostatic redox levels in cells (Driscoll and Copeland, 2003). It has been shown that the SeC-decoding complex, containing $tRNA^{SeC}$, is assembled onto specific mRNAs in the nucleus (de Jesus et al., 2006); therefore, $tRNA^{SeC}$ retrograde transport may promote synthesis of selenoproteins and protect cells from oxidative damage. Alternatively, accumulation of truncated $tRNA^{Sec}$ might contribute to the lowering of available $tRNA^{Sec}$ initiated by Brf2, a redox sensor that regulates $tRNA^{Sec}$ transcription in complex with transcription factor II B (TFIIB) (Gouge et al., 2015).

The observed induction of tRNA retrograde transport by MMS and puromycin also suggests that this pathway might protect human cells from stress. In yeast cells, certain tRNA modifications are important to ensure efficient translation of genes coding for DNA damage response proteins (Begley et al., 2007). Indeed, tRNA modification defects have been shown to make yeast cells more susceptible to MMS-induced cell death, establishing a link between DNA damage and tRNA availability (Begley et al., 2007). Puromycin, which acts as a non-functional mimic of aminoacyl tRNA, causes premature termination of translation, accumulation of aborted and improperly folded polypeptides, and activation of the UPR (Hightower, 1980; Neznanov et al., 2011). Therefore, our results support a unifying hypothesis (Figure 7E) whereby the selective tRNA nuclear accumulation we described might be part of a response that favors translation of certain stress-related genes

while reducing global translation to protect cells from damage. It will be interesting to understand how tRNA retrograde transport integrates with other tRNA-regulatory pathways that affect tRNA stability and function in response to stress (Roundtree et al., 2017). We note that stress-induced nuclear accumulation of cytoplasmic tRNAs has been recently observed in human cells using micro-injection techniques (Dhakal et al., 2018). Our results underscore the importance of small RNAs in protecting cells from oxidative stress, a phenomenon conserved from bacteria to humans (Storz, 2016).

STAR★METHODS

Detailed methods are provided in the online version of this paper and include the following:

- KEY RESOURCES TABLE
- CONTACT FOR REAGENT AND RESOURCE SHARING
- EXPERIMENTAL MODEL AND SUBJECT DETAILS
- METHOD DETAILS
  - Induction of cellular stress
  - Knockdown of EIF2AK4
  - Fluorescence *in situ* hybridization
  - Confocal Microscopy
  - Cell fractionation, Northern and western blotting
  - RNA and Sequencing Library preparation
  - NextSeq RNA sequencing and analysis
  - PCR assay for mTOR and UPR
- QUANTIFICATION AND STATISTICAL ANALYSIS
  - Quantificaion of nuclear:cytosolic fluorescence ratio
- DATA AND SOFTWARE AVAILABILITY

SUPPLEMENTAL INFORMATION

Supplemental Information can be found with this article online at https://doi.org/10.1016/j.celrep.2019.02.077.

ACKNOWLEDGMENTS

We thank K. Midwood (Oxford University) for the neo-NHDF cells, J. Brugarolas (UT Southwestern) for the REDD$^{-/-}$ MEFs, J. Kriston-Vizi (LMBC, UCL) for the ImageJ script, and D. Depledge for assistance with RNA-seq. We thank the Oxford Oncology Microscopy core for support with imaging. This study was funded by the BBSRC (BB/L021404/1); the Wellcome Trust (101849/Z/13/A); the European Union (ERC-2014-AdG-671231HEPCIR and H2020-667273-HEPCAR); ANRS; the French Cancer Agency (ARC IHU201301187); the IdEx Program of the University of Strasbourg; the Foundation University of Strasbourg; and the Department of Defense (W81XWH-16-1-0363). This work has been published under the framework of the LABEX ANR-10-LAB28 and benefits from funding from the state, managed by the French National Research Agency as part of the Investments for the Future Program.

AUTHOR CONTRIBUTIONS

H.S., F.J., M.M., and A.F. designed the experiments. H.S., L.J.P., and A.C. performed the experiments. F.J., H.S., T.F.B., and A.F. analyzed the data. A.F. and H.S. wrote the paper with input from M.M. and F.J.

DECLARATION OF INTERESTS

The authors declare no competing interests.

Received: August 29, 2018
Revised: February 4, 2019
Accepted: February 20, 2019
Published: March 19, 2019

REFERENCES

Ait Ghezala, H., Jolles, B., Salhi, S., Castrillo, K., Carpentier, W., Cagnard, N., Bruhat, A., Fafournoux, P., and Jean-Jean, O. (2012). Translation termination efficiency modulates ATF4 response by regulating ATF4 mRNA translation at 5′ short ORFs. Nucleic Acids Res. *40*, 9557–9570.

Akira, S., Hirano, T., Taga, T., and Kishimoto, T. (1990). Biology of multifunctional cytokines: IL 6 and related molecules (IL 1 and TNF). FASEB J. *4*, 2860–2867.

Axten, J.M., Medina, J.R., Feng, Y., Shu, A., Romeril, S.P., Grant, S.W., Li, W.H., Heerding, D.A., Minthorn, E., Mencken, T., et al. (2012). Discovery of 7-methyl-5-(1-{[3-(trifluoromethyl)phenyl]acetyl}-2,3-dihydro-1H-indol-5-yl)-7H-p yrrolo[2,3-d]pyrimidin-4-amine (GSK2606414), a potent and selective first-in-class inhibitor of protein kinase R (PKR)-like endoplasmic reticulum kinase (PERK). J. Med. Chem. *55*, 7193–7207.

Barhoom, S., Kaur, J., Cooperman, B.S., Smorodinsky, N.I., Smilansky, Z., Ehrlich, M., and Elroy-Stein, O. (2011). Quantitative single cell monitoring of protein synthesis at subcellular resolution using fluorescently labeled tRNA. Nucleic Acids Res. *39*, e129.

Begley, U., Dyavaiah, M., Patil, A., Rooney, J.P., DiRenzo, D., Young, C.M., Conklin, D.S., Zitomer, R.S., and Begley, T.J. (2007). Trm9-catalyzed tRNA modifications link translation to the DNA damage response. Mol. Cell *28*, 860–870.

Boccaletto, P., Machnicka, M.A., Purta, E., Piatkowski, P., Baginski, B., Wirecki, T.K., de Crécy-Lagard, V., Ross, R., Limbach, P.A., Kotter, A., et al. (2018). MODOMICS: a database of RNA modification pathways. 2017 update. Nucleic Acids Res. *46* (*D1*), D303–D307.

Brugarolas, J., Lei, K., Hurley, R.L., Manning, B.D., Reiling, J.H., Hafen, E., Witters, L.A., Ellisen, L.W., and Kaelin, W.G., Jr. (2004). Regulation of mTOR function in response to hypoxia by REDD1 and the TSC1/TSC2 tumor suppressor complex. Genes Dev. *18*, 2893–2904.

Chafe, S.C., Pierce, J.B., Eswara, M.B., McGuire, A.T., and Mangroo, D. (2011). Nutrient stress does not cause retrograde transport of cytoplasmic tRNA to the nucleus in evolutionarily diverse organisms. Mol. Biol. Cell *22*, 1091–1103.

Chan, C.T., Dyavaiah, M., DeMott, M.S., Taghizadeh, K., Dedon, P.C., and Begley, T.J. (2010). A quantitative systems approach reveals dynamic control of tRNA modifications during cellular stress. PLoS Genet. *6*, e1001247.

Chatterjee, K., Majumder, S., Wan, Y., Shah, V., Wu, J., Huang, H.Y., and Hopper, A.K. (2017). Sharing the load: Mex67-Mtr2 cofunctions with Los1 in primary tRNA nuclear export. Genes Dev. *31*, 2186–2198.

Chu, H.Y., and Hopper, A.K. (2013). Genome-wide investigation of the role of the tRNA nuclear-cytoplasmic trafficking pathway in regulation of the yeast Saccharomyces cerevisiae transcriptome and proteome. Mol. Cell. Biol. *33*, 4241–4254.

Clark, W.C., Evans, M.E., Dominissini, D., Zheng, G., and Pan, T. (2016). tRNA base methylation identification and quantification via high-throughput sequencing. RNA *22*, 1771–1784.

Couvillion, M.T., Sachidanandam, R., and Collins, K. (2010). A growth-essential Tetrahymena Piwi protein carries tRNA fragment cargo. Genes Dev. *24*, 2742–2747.

Cubillos-Ruiz, J.R., Bettigole, S.E., and Glimcher, L.H. (2017). Tumorigenic and Immunosuppressive Effects of Endoplasmic Reticulum Stress in Cancer. Cell *168*, 692–706.

Czech, A., Wende, S., Mörl, M., Pan, T., and Ignatova, Z. (2013). Reversible and rapid transfer-RNA deactivation as a mechanism of translational repression in stress. PLoS Genet. *9*, e1003767.

de Jesus, L.A., Hoffmann, P.R., Michaud, T., Forry, E.P., Small-Howard, A., Stillwell, R.J., Morozova, N., Harney, J.W., and Berry, M.J. (2006). Nuclear

assembly of UGA decoding complexes on selenoprotein mRNAs: a mechanism for eluding nonsense-mediated decay? Mol. Cell. Biol. *26*, 1795–1805.

Dennis, M.D., McGhee, N.K., Jefferson, L.S., and Kimball, S.R. (2013). Regulated in DNA damage and development 1 (REDD1) promotes cell survival during serum deprivation by sustaining repression of signaling through the mechanistic target of rapamycin in complex 1 (mTORC1). Cell. Signal. *25*, 2709–2716.

Dhakal, R., Tong, C., Anderson, S., Kashina, A.S., Cooperman, B., and Bau, H.H. (2018). Dynamics of intracellular stress-induced tRNA trafficking. Nucleic Acids Res. Published online November 28, 2018. https://doi.org/10.1093/nar/gky1208.

Driscoll, D.M., and Copeland, P.R. (2003). Mechanism and regulation of selenoprotein synthesis. Annu. Rev. Nutr. *23*, 17–40.

Fu, H., Feng, J., Liu, Q., Sun, F., Tie, Y., Zhu, J., Xing, R., Sun, Z., and Zheng, X. (2009). Stress induces tRNA cleavage by angiogenin in mammalian cells. FEBS Lett. *583*, 437–442.

Gebauer, F., and Hentze, M.W. (2004). Molecular mechanisms of translational control. Nat. Rev. Mol. Cell Biol. *5*, 827–835.

Gerbi, S.A., Borovjagin, A.V., Odreman, F.E., and Lange, T.S. (2003). U4 snRNA nucleolar localization requires the NHPX/15.5-kD protein binding site but not Sm protein or U6 snRNA association. J. Cell Biol. *162*, 821–832.

Gouge, J., Satia, K., Guthertz, N., Widya, M., Thompson, A.J., Cousin, P., Dergai, O., Hernandez, N., and Vannini, A. (2015). Redox Signaling by the RNA Polymerase III TFIIB-Related Factor Brf2. Cell *163*, 1375–1387.

Harding, H.P., Novoa, I., Zhang, Y., Zeng, H., Wek, R., Schapira, M., and Ron, D. (2000). Regulated translation initiation controls stress-induced gene expression in mammalian cells. Mol. Cell *6*, 1099–1108.

Harding, H.P., Calfon, M., Urano, F., Novoa, I., and Ron, D. (2002). Transcriptional and translational control in the Mammalian unfolded protein response. Annu. Rev. Cell Dev. Biol. *18*, 575–599.

Harding, H.P., Zhang, Y., Zeng, H., Novoa, I., Lu, P.D., Calfon, M., Sadri, N., Yun, C., Popko, B., Paules, R., et al. (2003). An integrated stress response regulates amino acid metabolism and resistance to oxidative stress. Mol. Cell *11*, 619–633.

Hightower, L.E. (1980). Cultured animal cells exposed to amino acid analogues or puromycin rapidly synthesize several polypeptides. J. Cell. Physiol. *102*, 407–427.

Hopper, A.K. (2013). Transfer RNA post-transcriptional processing, turnover, and subcellular dynamics in the yeast Saccharomyces cerevisiae. Genetics *194*, 43–67.

Huang, H.Y., and Hopper, A.K. (2014). Separate responses of karyopherins to glucose and amino acid availability regulate nucleocytoplasmic transport. Mol. Biol. Cell *25*, 2840–2852.

Huang, H.Y., and Hopper, A.K. (2016). Multiple Layers of Stress-Induced Regulation in tRNA Biology. Life (Basel) *6*, E16.

Hurto, R.L., Tong, A.H., Boone, C., and Hopper, A.K. (2007). Inorganic phosphate deprivation causes tRNA nuclear accumulation via retrograde transport in Saccharomyces cerevisiae. Genetics *176*, 841–852.

Ivanov, P., Emara, M.M., Villen, J., Gygi, S.P., and Anderson, P. (2011). Angiogenin-induced tRNA fragments inhibit translation initiation. Mol. Cell *43*, 613–623.

Jammi, N.V., Whitby, L.R., and Beal, P.A. (2003). Small molecule inhibitors of the RNA-dependent protein kinase. Biochem. Biophys. Res. Commun. *308*, 50–57.

Kim, D., Langmead, B., and Salzberg, S.L. (2015). HISAT: a fast spliced aligner with low memory requirements. Nat. Methods *12*, 357–360.

Kramer, E.B., and Hopper, A.K. (2013). Retrograde transfer RNA nuclear import provides a new level of tRNA quality control in Saccharomyces cerevisiae. Proc. Natl. Acad. Sci. USA *110*, 21042–21047.

Kroemer, G., Mariño, G., and Levine, B. (2010). Autophagy and the integrated stress response. Mol. Cell *40*, 280–293.

Kuwano, Y., Rabinovic, A., Srikantan, S., Gorospe, M., and Demple, B. (2009). Analysis of nitric oxide-stabilized mRNAs in human fibroblasts reveals HuR-dependent heme oxygenase 1 upregulation. Mol. Cell. Biol. *29*, 2622–2635.

Levy, D., Larner, A., Chaudhuri, A., Babiss, L.E., and Darnell, J.E., Jr. (1986). Interferon-stimulated transcription: isolation of an inducible gene and identification of its regulatory region. Proc. Natl. Acad. Sci. USA *83*, 8929–8933.

Love, M.I., Huber, W., and Anders, S. (2014). Moderated estimation of fold change and dispersion for RNA-seq data with DESeq2. Genome Biol. *15*, 550.

Lowe, T.M., and Chan, P.P. (2016). tRNAscan-SE On-line: integrating search and context for analysis of transfer RNA genes. Nucleic Acids Res. *44* (*W1*), W54–W57.

Martin, M.M. (2011). Cutadapt removes adapter sequences from high-throughput sequencing reads. EMBnet.journal *17*, 10–12.

Miyagawa, R., Mizuno, R., Watanabe, K., and Ijiri, K. (2012). Formation of tRNA granules in the nucleus of heat-induced human cells. Biochem. Biophys. Res. Commun. *418*, 149–155.

Murthi, A., Shaheen, H.H., Huang, H.Y., Preston, M.A., Lai, T.P., Phizicky, E.M., and Hopper, A.K. (2009). Regulation of tRNA Bidirectional Nuclear-Cytoplasmic Trafficking in S. cerevisiae. Mol. Biol. Cell *21*, 639–649.

Neznanov, N., Komarov, A.P., Neznanova, L., Stanhope-Baker, P., and Gudkov, A.V. (2011). Proteotoxic stress targeted therapy (PSTT): induction of protein misfolding enhances the antitumor effect of the proteasome inhibitor bortezomib. Oncotarget *2*, 209–221.

Nottingham, R.M., Wu, D.C., Qin, Y., Yao, J., Hunicke-Smith, S., and Lambowitz, A.M. (2016). RNA-seq of human reference RNA samples using a thermostable group II intron reverse transcriptase. RNA *22*, 597–613.

Ohira, T., and Suzuki, T. (2011). Retrograde nuclear import of tRNA precursors is required for modified base biogenesis in yeast. Proc. Natl. Acad. Sci. USA *108*, 10502–10507.

Pakos-Zebrucka, K., Koryga, I., Mnich, K., Ljujic, M., Samali, A., and Gorman, A.M. (2016). The integrated stress response. EMBO Rep. *17*, 1374–1395.

Paushkin, S.V., Patel, M., Furia, B.S., Peltz, S.W., and Trotta, C.R. (2004). Identification of a human endonuclease complex reveals a link between tRNA splicing and pre-mRNA 3′ end formation. Cell *117*, 311–321.

Qin, Y., Yao, J., Wu, D.C., Nottingham, R.M., Mohr, S., Hunicke-Smith, S., and Lambowitz, A.M. (2016). High-throughput sequencing of human plasma RNA by using thermostable group II intron reverse transcriptases. RNA *22*, 111–128.

R Development Core Team (2011). R: A language and environment for statistical computing. R Foundation for Statistical Computing, Vienna, Austria.http://www.R-project.org.

Roundtree, I.A., Evans, M.E., Pan, T., and He, C. (2017). Dynamic RNA Modifications in Gene Expression Regulation. Cell *169*, 1187–1200.

Sadler, A.J., and Williams, B.R. (2008). Interferon-inducible antiviral effectors. Nat. Rev. Immunol. *8*, 559–568.

Sarkar, S., and Hopper, A.K. (1998). tRNA nuclear export in saccharomyces cerevisiae: in situ hybridization analysis. Mol. Biol. Cell *9*, 3041–3055.

Saxton, R.A., and Sabatini, D.M. (2017). mTOR Signaling in Growth, Metabolism, and Disease. Cell *169*, 361–371.

Schieber, M., and Chandel, N.S. (2014). ROS function in redox signaling and oxidative stress. Curr. Biol. *24*, R453–R462.

Sengupta, S., Peterson, T.R., and Sabatini, D.M. (2010). Regulation of the mTOR complex 1 pathway by nutrients, growth factors, and stress. Mol. Cell *40*, 310–322.

Shaheen, H.H., and Hopper, A.K. (2005). Retrograde movement of tRNAs from the cytoplasm to the nucleus in Saccharomyces cerevisiae. Proc. Natl. Acad. Sci. USA *102*, 11290–11295.

Shaheen, H.H., Horetsky, R.L., Kimball, S.R., Murthi, A., Jefferson, L.S., and Hopper, A.K. (2007). Retrograde nuclear accumulation of cytoplasmic tRNA in rat hepatoma cells in response to amino acid deprivation. Proc. Natl. Acad. Sci. USA *104*, 8845–8850.

Shoshani, T., Faerman, A., Mett, I., Zelin, E., Tenne, T., Gorodin, S., Moshel, Y., Elbaz, S., Budanov, A., Chajut, A., et al. (2002). Identification of a novel hypoxia-inducible factor 1-responsive gene, RTP801, involved in apoptosis. Mol. Cell. Biol. *22*, 2283–2293.

Söll, D., and RajBhandary, U. (1995). tRNA: Structure, Biosynthesis, and Function (ASM Press).

Spriggs, K.A., Bushell, M., and Willis, A.E. (2010). Translational regulation of gene expression during conditions of cell stress. Mol. Cell *40*, 228–237.

Storz, G. (2016). New perspectives: Insights into oxidative stress from bacterial studies. Arch. Biochem. Biophys. *595*, 25–27.

Takano, A., Endo, T., and Yoshihisa, T. (2005). tRNA actively shuttles between the nucleus and cytosol in yeast. Science *309*, 140–142.

Takano, A., Kajita, T., Mochizuki, M., Endo, T., and Yoshihisa, T. (2015). Cytosolic Hsp70 and co-chaperones constitute a novel system for tRNA import into the nucleus. eLife *4*, 4.

Thompson, D.M., Lu, C., Green, P.J., and Parker, R. (2008). tRNA cleavage is a conserved response to oxidative stress in eukaryotes. RNA *14*, 2095–2103.

Wang, M., and Kaufman, R.J. (2014). The impact of the endoplasmic reticulum protein-folding environment on cancer development. Nat. Rev. Cancer *14*, 581–597.

Watanabe, K., Miyagawa, R., Tomikawa, C., Mizuno, R., Takahashi, A., Hori, H., and Ijiri, K. (2013). Degradation of initiator tRNAMet by Xrn1/2 via its accumulation in the nucleus of heat-treated HeLa cells. Nucleic Acids Res. *41*, 4671–4685.

Wellner, K., Betat, H., and Mörl, M. (2018). A tRNA's fate is decided at its 3′ end: Collaborative actions of CCA-adding enzyme and RNases involved in tRNA processing and degradation. Biochim. Biophys. Acta. Gene Regul. Mech. *1861*, 433–441.

Whitney, M.L., Hurto, R.L., Shaheen, H.H., and Hopper, A.K. (2007). Rapid and reversible nuclear accumulation of cytoplasmic tRNA in response to nutrient availability. Mol. Biol. Cell *18*, 2678–2686.

Whitney, M.L., Jefferson, L.S., and Kimball, S.R. (2009). ATF4 is necessary and sufficient for ER stress-induced upregulation of REDD1 expression. Biochem. Biophys. Res. Commun. *379*, 451–455.

Winterbourn, C.C., and Hampton, M.B. (2008). Thiol chemistry and specificity in redox signaling. Free Radic. Biol. Med. *45*, 549–561.

Woo, K.J., Lee, T.J., Park, J.W., and Kwon, T.K. (2006). Desferrioxamine, an iron chelator, enhances HIF-1alpha accumulation via cyclooxygenase-2 signaling pathway. Biochem. Biophys. Res. Commun. *343*, 8–14.

Wu, J., Huang, H.Y., and Hopper, A.K. (2013). A rapid and sensitive non-radioactive method applicable for genome-wide analysis of Saccharomyces cerevisiae genes involved in small RNA biology. Yeast *30*, 119–128.

Yamasaki, S., Ivanov, P., Hu, G.F., and Anderson, P. (2009). Angiogenin cleaves tRNA and promotes stress-induced translational repression. J. Cell Biol. *185*, 35–42.

Yoshihisa, T., Yunoki-Esaki, K., Ohshima, C., Tanaka, N., and Endo, T. (2003). Possibility of cytoplasmic pre-tRNA splicing: the yeast tRNA splicing endonuclease mainly localizes on the mitochondria. Mol. Biol. Cell *14*, 3266–3279.

Zaitseva, L., Myers, R., and Fassati, A. (2006). tRNAs promote nuclear import of HIV-1 intracellular reverse transcription complexes. PLoS Biol. *4*, e332.

Zhai, Y., Zhong, Z., Chen, C.Y., Xia, Z., Song, L., Blackburn, M.R., and Shyu, A.B. (2008). Coordinated changes in mRNA turnover, translation, and RNA processing bodies in bronchial epithelial cells following inflammatory stimulation. Mol. Cell. Biol. *28*, 7414–7426.

Zhou, K.I., Clark, W.C., Pan, D.W., Eckwahl, M.J., Dai, Q., and Pan, T. (2018). Pseudouridines have context-dependent mutation and stop rates in high-throughput sequencing. RNA Biol. *15*, 892–900.

STAR★METHODS

KEY RESOURCES TABLE

REAGENT or RESOURCE	SOURCE	IDENTIFIER
Antibodies		
Rabbit Antibody against *GAPDH*	Sigma	Cat# G9545; RRID: AB_796208
Rabbit Antibody against lamin B1	Life Technologies	Cat# PA5-19468; RRID: AB_10985414
Rabbit Antibody against PDI	Cell Signaling Technology	Cat# mAb3501; RRID: AB
Rabbit Antibody against REDD1/DDIT4	Novus Biologicals	Cat# NBP1-77321SS; RRID: AB_11036185
Rabbit Antibody against Phospho-eIF2 alpha	Sigma	Cat# SAB4504388
Rabbit Antibody against 4EBP1	Bethyl Laboratories	Cat# A300-501; RRID: AB_2277825
Rabbit Antibody against HIF-1	Abcam	Cat# Ab51608; RRID: AB_880418
Mouse mAb against S6 Ribosomal Protein	Cell Signaling Technology	Cat# 2317; RRID: AB_2238583
Rabbit Antibody against Phospho-S6 Ribosomal Protein (Ser235/236)	Cell Signaling Technology	Cat# 4858S; RRID: AB_916156
Rabbit Antibody against Actin	Sigma	Cat# A2066; RRIB: AB_476693
Rabbit Antibody against Na/K ATPase	Santa Cruz	Cat# SC58626; RRIB: AB_781529
DIG specific FAB fragments (Fluorescein)	Roche	Cat# 11207741910; RRID: AB_514498
Goat anti-rabbit antibody (HRP)	Dako	Cat# P0448; RRID: AB_2617138
Goat anti-Rabbit IgG H&L (IRDye® 800CW) preadsorbed	Abcam	Cat# Ab216773
Goat anti mouse	Dako	Cat# P0447; RIID: AB_2617137
Digoxigenin Antibody (9H27L19)	Thermo Fisher	Cat# 700772; RIID: AB_1024571
Biological Samples		
T cells, peripheral blood	healthy volunteers	N/A
Chemicals, Peptides, and Recombinant Proteins		
Recombinant IFNα	Thermo Fisher	Cat# PHC4014
Recombinant TNFα	Thermo Fisher	Cat# PHC3015
Actinomycin D	Sigma	Cat# A1410
Ficoll-Hypaque Plus	GE Healthcare	Cat# GE17-1440-02
Imidazolo-oxindole PKR inhibitor C16	Sigma	Cat# I9785
Desferrioxamine	Sigma	Cat# D9533
EIF2AK4 (ID 440275) Trilencer-27 Human siRNA	Origene	Cat# SR318498
GSK2606414	Merck	Cat# 516535
ER Tracker^{TM} Red	Thermo Fisher	Cat# E34250
deionized Formamide	Sigma	Cat# F9037
Dextran	Sigma	Cat# D8906
Hering Sperm DNA	Invitrogen	Cat#15634017
ProLong Gold Antifade Mountant with DAPI	Molecular Probes	Cat# P36931
Nuclei Ez Prep kit	Sigma	Cat# NUC101
TRI Reagent	Sigma	Cat# T9424
Agencurt AMPure XP beads	Beckman	Cat# A63880
Critical Commercial Assays		
TACS Annexin V-FITC Apoptosis Detection Kit	R&D	Cat# 4830-250-K
Pan T cell isolation kit	Miltenyi Biotec	Cat# 130-096-535
High-Capacity cDNA Reverse Transcription Kit	Applied Bioscience	Cat# 4368814
QuantiTect SYBR Green RT-PCR Kit	QIAGEN	Cat# 204243
PureLink® miRNA Isolation Kit	Ambion	Cat# K157001

(*Continued on next page*)

Continued

REAGENT or RESOURCE	SOURCE	IDENTIFIER
TGIRT Template-Switching RNA-seq Kit	InGex	Cat #TGIRTKit25
5′DNA Adenylation Kit	NEB	Cat# E2610S
Thermostable 5′ AppDNA/RNA Ligase	NEB	Cat# M0319S
Phusion High Fidality PCR Master Mix	Life Technologies	Cat# F-531S
NextSeq® 500/550 Mid Output Kit v2 (150 cycles)	Illumina	Cat# FC-404-2001
mTOR SAB target list H96 qPCR set	BioRad	N/A
Unfolded protein response SAB target list qPCR set	BioRad	N/A
Deposited Data		
TGRIT sequencing data	BioProject NCBI	submission ID: SUB2812477, BioProject NCBI: PRJNA391929
Experimental Models: Cell Lines		
Neo-NHDFs (neonatal Normal Human Dermal Fibroblasts)	Gift from Kim Midwood	N/A
HeLa	Gift from Clare Jolly	N/A
Oligonucleotides		
Please see Table S7		
Software and Algorithms		
cutadapt v1.11	https://cutadapt.readthedocs.io/	Martin, 2011
hisat2 v2.0.4	https://ccb.jhu.edu/software/hisat2/index.shtml	Kim et al., 2015
R	https://www.r-project.org/	R Development Core Team, 2011
DESeq2	https://bioconductor.org/packages/release/bioc/html/DESeq2.html	Love et al., 2014
gplots package	https://cran.r-project.org/web/packages/gplots/gplots.pdf	N/A
R library ggplots2	https://cran.r-project.org/web/packages/ggplot2/index.html	N/A
GraphPad Prism 7.01	https://www.graphpad.com/scientific-software/prism/	N/A
ImageJ 1.49	https://imagej.nih.gov/ij/	N/A
Other		
GtRNAdb 2.0	http://gtrnadb.ucsc.edu/	Lowe and Chan 2016
Ensembl ncRNA compilation (GRCh37/75)	http://ftp.ensembl.org/pub/release-75/fasta/homo_sapiens/dna/	N/A

CONTACT FOR REAGENT AND RESOURCE SHARING

Further information and requests for resources and reagents should be directed to and will be fulfilled by the Lead Contact, Ariberto Fassati (a.fassati@ucl.ac.uk).

EXPERIMENTAL MODEL AND SUBJECT DETAILS

Neo-NHDFs (neonatal Normal Human Dermal Fibroblasts) (male) and HeLa cells (female) were cultured in Dulbecco’s Modified Eagle Medium (DMEM) (GIBCO) supplemented with 10% FCS (Fetal Calf Serum, Labtech), 100 units/ml penicillin and 100 μg/ml streptomycin at 37°C, 5.0% CO_2. For primary human T cell isolation, peripheral blood was obtained from healthy volunteers (one male and one female). All participants donating blood for this study gave written informed consent. Ethical approval was given by the South East Coast Research Ethics Committee under the REC reference number: 11/LO/0421 and IRAS project number: 43993. Mononuclear cells (PBMC) were isolated from fresh heparinised blood by density centrifugation using Ficoll-Hypaque Plus (GE Healthcare). CD3+ T cells were subsequently isolated from PBMC using magnetic bead isolation (Pan T cell isolation kit; Miltenyi Biotec) as per the manufacturer’s instructions.

METHOD DETAILS

Induction of cellular stress

T cells were used 2 hours after isolation, HeLa cells and neo-NHDFs were plated at 70 - 80% confluence and 16 to 24 hours later media was changed to fresh media containing H_2O_2 at the indicated concentrations, or IFNα (1000 U/ml), or TNFα (10ng/ml) or glucose-free media. For heat shock, cells were incubated in a humidified incubator at 42°C for up to 1 hour. The pH of DMEM media was adjusted with 0.1% v/v from 1M HCl stock or 0.1% v/v from 1M NaOH stock. At the indicated time post-treatment, cells were washed once in phosphate buffered saline (PBS) and processed for tFISH or fractionation. To monitor viability, cells were stained for annexin and PI using the TACS Annexin V-FITC Apoptosis Detection Kit (R&D systems) following the manufacturer's instructions and analyzed by flow cytometry.

Knockdown of EIF2AK4

For transient knockdown of mRNA encoding EIF2AK4, cells were transfected with Trilencer-27 Human siRNA using Oligofectamin accordingly to the manufacturer's instructions. Briefly, for a 6-well plate transfection format, 10 µL transfection reagent and 20 µL Optimem Medium were pre-mixed. Subsequently, 161 µL Optimem and 3 µL of each siRNA oligo were added. After 20 min incubation at room temperature, 800 µL of Optimem was added and cells were transfected with 1ml transfection mixture. Four hours post-transfection, 1 mL DMEM supplemented with 20% FCS (Fetal Calf Serum, Labtech), 100 units/ml penicillin and 100 µg/ml streptomycin was added. 48 hours post-transfection, cells were split into either 6-well plates or 12-well plates for RNA extraction and tFISH analysis, respectively. Experiments were performed 72 h post-transfection.

Fluorescence *in situ* hybridization

The Fluorescence *in situ* hybridization method was adapted from Sarkar and Hopper (1998). Briefly, the day before the experiment, cells were plated into 12-well plates containing glass coverslips (VWR), which were coated with 0.0025% poly-L-lysine (Sigma-Aldrich) for experiments with neo-NHDFs. Following exposure to the indicated condition, cells were washed with PBS and subsequently fixed with freshly prepared 4% Paraformaldehyde in PBS at 25°C for 50 minutes. The reaction was quenched with 100 mM Glycine in PBS. For ER detection, cells were incubated with ER Tracker™ Red (Molecular Probes) following the manufacturer's instructions. Subsequently, cells were permeabilized with ice-cold acetone at −20°C for 3 minutes and washed three times with PBS. Coverslips were incubated for 10 minutes in pre-hybridization buffer (4x SSC, 3% BSA and 50% deionized Formamide [Sigma]) at 25°C. Hybridization was performed overnight at 37°C in 4x SSC, 0.2% BSA, 50% deionized Formamide, 10% Dextran (Sigma), 160 µg/ml Hering Sperm DNA (Invitrogen) and 0.7 ng/µl DIG-labeled oligonucleotides. After hybridization, cells were washed sequentially in 4x, 2x and 0,5x SSC and 50% deionized Formamide. For antibody detection of Dig labeled probes, cells were blocked with 3% BSA, 2xSSC and 0.1% Triton-100 for 2 hours at 25°C. Subsequently, DIG-labeled probes were detected with DIG specific FAB fragments (Roche) conjugated to Fluorescein. Samples were washed 4 times in 2x SSC and 0.1% Triton. Coverslips were mounted on microscopic slides with ProLong Gold Antifade Mountant with DAPI (Molecular Probes).

Confocal Microscopy

Confocal images were acquired at the Confocal Imaging Unit (UCL Department of Cell and Developmental Biology). Single stack images were acquired on a Leica TCS SPE inverted Microscope using a 40x or 63x objective with a resolution of 1024x1024 pixels under sequentially imaging settings. DAPI and Fluorescein fluorescence emission were detected with 405nm and 488nm lasers respectively using the manufactured emission wavelength setting for DAPI and Alexa Fluor 488. Settings were adjusted for each experiment accordingly to the quality of stain but retained for all samples within the same experiment. Images were processed using LAS-X software and analyzed using ImageJ software package. Fluorescence intensities were determined after automated segregation of nuclear and cytosolic area using DAPI as nuclear stain (ImageJ macro kindly provided by Janos Kriston-Vizi, UCL).

Cell fractionation, Northern and western blotting

Cells were grown in 10cm tissue culture plates and fractionated using the Nuclei Ez Prep kit (Sigma) following the manufacturer's instructions. Aliquots of 10% volume were used for western blotting and the RNA was extracted from the remaining cell lysate or fractionated lysates using Trizol. For Northern blot, 10-15 µg of purified RNA were loaded onto a 12% polyacrylamide denaturing gel containing 8M Urea in 1xTBE. Following PAGE, RNA was transferred to a positively charged nylon membrane (GE Healthcare) and crosslinked to the membrane using a conventional UV trans-illuminator. Membrane was pre-hybridized for 30 minutes at 37°C with 5xSSC, 0.1% N-Lauroyslsacrosine and 0.02% SDS, 1% Blocking reagent (Roche). Subsequently, the membrane was incubated overnight at 37°C in pre-hybridization buffer containing 5 nM heat denatured DIG-labeled probes (Wu et al., 2013). The membrane was washed 4 times for 10 minutes each in 2xSSC and 0.1% SDS. For detection of DIG-labeled probes, the membrane was briefly washed in washing buffer (0.1M Maleic Acid, 0.15M NaCl, pH 7,5 and 0.3% Tween, pH7.5) and blocked with 0.1M Maleic Acid, 0.15M NaCl, 1% Blocking Reagent (Roche) at room temperature. Primary anti-DIG antibody (700772, Life Technology) and HRP-conjugated goat anti-rabbit antibody were diluted in 0.1M Maleic Acid, 0.15M NaCl and subsequently incubated with the membrane for 1 hour at room temperature then the membrane was washed 2 times for 15 minutes each with washing buffer. Detection

was with ECL Prime Western Blotting Detection Reagent (GE Healtcare Life Science) and Hyperfilm ECL (GE Healtcare Life Science). For western blot, 1/50 of total lysates volume was loaded onto a 10% SDS-PAGE and transferred to an Immobilon-P PVDF membrane (Millipore) followed by incubation with primary and HRP-conjugated secondary antibodies and ECL detection. Inhibition of *de novo* RNA transcription by ActD was validated by quantitative PCR. 1 μg total RNA was reverse transcribed using the High-Capacity cDNA Reverse Transcription Kit (Applied Biosystems) following the manufacturer's instructions. cDNAs were quantified on a Eppendorf Masterplex thermocycler using QuantiTect SYBR Green RT-PCR Kit (QIAGEN). Primer sequences are detailed in the Key Resources Table. Ct values were normalized to the housekeeping gene *GAPDH*.

RNA and Sequencing Library preparation

For NextSeq RNA sequencing, small RNAs were enriched using the PureLink® miRNA Isolation Kit (Ambion^{TM}). The RNA library preparation was adapted from Nottingham et al. (2016) and Qin et al. (2016). Briefly, 2 μg of small-size RNA (< 200 nt) were deacylated with 0.1 mM TrisHCl pH = 9 at 37°C for 30 min in the presence of RNasin Ribonuclease Inhibitor (Promega). Potential 3′ phosphates were removed by incubation at 37°C for 45 minutes using T4 Polynucleotide Kinase (Promega). RNA was purified using Chloroform extraction and EtOH precipitation. RNA libraries were generated from 100ng treated small RNA using TGIRT^{TM}- III Enzyme (InGex, USA) using the manufacturer's protocol for the total RNA-seq method. Sequencing libraries were amplified for 15 cycles using Phusion High-Fidelity PCR Master Mix and purified using 1.3-1.4xAgentcourt AMPure XP beads. The quality of the RNA libraries was analyzed using Qubit System and TapeStation. Libraries were sequenced on an Illumina NextSeq 550 Instrument at the UCL Pathogen Genomics Unit (London).

NextSeq RNA sequencing and analysis

Primer were clipped from raw read data using cutadapt v1.11 (Martin, 2011). The sequences of the complete set of human tRNA genes were downloaded from GtRNAdb (Lowe and Chan, 2016) and pseudogenes were removed. This set was merged with the Ensembl ncRNA compilation (GRCh37/75). Clipped reads were mapped to this comprehensive set of ncRNAs including human tRNAs and all other ncRNAs using hisat2 v2.0.4 (Kim et al., 2015). Uniquely mapped reads were counted for every gene, and differentially expressed genes were analyzed using R (R Development Core Team, 2011) and DESeq2 (Love et al., 2014). Clustering of intact and defective tRNA were calculated with the R function heatmap.2 of the gplots package. Figures were created using the R library ggplots2 and GraphPad Prism 7.

PCR assay for mTOR and UPR

HeLa cells were seeded in 6-well plates and grown overnight to 70% confluency. Next day the media was replaced and cells were treated with 5 mM H_2O_2 for 2.15 hours. Cells were washed with PBS and RNA was extracted using Trizol. One μg of total RNA was reverse transcribed using the High-Capacity cDNA Reverse Transcription Kit (Applied Biosystems) following the manufacturer's instructions. Expression profiling was performed according to the manufacturer's instructions using PrimePCR collection assays for the mTOR signaling (SAB Target List) H96 and Unfolded protein response (SAB Target List) H96 from BioRad. Ct values have been normalized against *TBP*, *GAPDH* and *HPRT1*. The experiment has been performed using three biological replicates. Data were analyzed using the PrimerPCR Analyis software from Bio-Rad (V1.0.030.1023).

QUANTIFICATION AND STATISTICAL ANALYSIS

Quantificaion of nuclear:cytosolic fluorescence ratio

Segregation of cytosolic and nuclear fluorescence signal were done on ImgageJ using subsequently following Macros (kindly provided by Janos Kriston-Vizi, UCL) and manually corrected. Approximately 100 cells were counted from at least 5 randomly chosen images per condition. Briefly, images were manually corrected for background and cells were separated using the freehand line tool of ImageJ. The DAPI staining was used to determine the area of the nucleus. For HeLa, T cells and Jurkat cells the nuclear and cytosolic segregation macros were applied and the mean fluorescence intensity calculated for the cytosolic (C) and nuclear (N) areas. tRNA subcellular localization is represented by the nuclear/cytoplasmic (N/C) ratio. For NHDF cells, nuclear (Nuc) and total cell (Cyt) raw fluorescence intensities (RID) were manually determined. The corresponding N/C ratio was calculated for each cell using the following equation:

$$N/C = \frac{\frac{RID_{Nuc.}}{Area_{Nuc.}}}{\frac{(RID_{Cyt.} - RID_{Nuc.})}{(Area_{Cyt.} - Area_{Nuc.})}}$$

The calculated N/C ratios were subsequently plotted using the GraphPad Prism software program (GraphPad Software, Inc.).

Nuclear segregation macro

```
run(“Properties…,” “channels=1 slices=1 frames=1 unit=pixel pixel_width=1 pixel_height=1 voxel_depth=1.0000000”);
imagetitle = getTitle();
```

```
rename("bin");
run("Split Channels");
run("Properties...," "channels=1 slices=1 frames=1 unit=pixel pixel_width=1 pixel_height=1 voxel_depth=1");
selectWindow("bin (blue)");
setAutoThreshold("Otsu dark");
setOption("BlackBackground," true);
run("Convert to Mask");
run("Dilate");
run("Fill Holes");
run("Set Measurements...," "mean standard median display redirect=[bin (green)] decimal=2");
run("ROI Manager...");
run("Analyze Particles...," "size=500-Infinity display exclude clear add");
Cytosolic segregation macro:
run("Set Measurements...," "mean standard median display redirect=[bin (green)] decimal=2");
setForegroundColor(0,0,0);
// roiManager("Deselect");
// roiManager("Delete");
selectWindow("bin (green)");
run("Duplicate...," "title=[bin (green)_blank]");
selectWindow("bin (blue)");
run("Create Selection");
selectWindow("bin (green)_blank");
run("Restore Selection");
run("Fill," "slice");
run("Select None");
setAutoThreshold("Otsu dark");
run("Convert to Mask");
run("Median...," "radius=2");
run("BinaryDilateNoMerge8 ", "iterations=2 white"); // Install BinaryDilateNoMerge8_.class from http://www.mecourse.com/landinig/software/software.html
selectWindow("bin (blue)");
run("Select None");
run("Duplicate...," "title=[NucRing]");
run("BinaryDilateNoMerge8 ", "iterations=2 white");
selectWindow("bin (blue)");
run("Create Selection");
selectWindow("NucRing");
run("Restore Selection");
run("Fill," "slice");
run("Select None");
imageCalculator("Add create," "bin (green)_blank,""NucRing");
selectWindow("Result of bin (green)_blank");
run("Analyze Particles...," "size=500-Infinity display exclude clear add");
```

Statistical analysis

One-way Anova (Dunnett's multiple comparisons test) was used to calculate statistical significance *$p = 0.05$; **$p < 0.05$; ***$p < 0.001$, ****$p < 0.0001$ between the means of multiple independent groups. Unpaired two-tailed Student's t test was performed to compare results from H_2O_2 induced sample to control as indicated in the figure legends. A p value < 0.05 was deemed statistically significant.

DATA AND SOFTWARE AVAILABILITY

The TGRIT sequencing data have been deposited at BioProject NCBI, submission ID: SUB2812477, BioProject NCBI: PRJNA391929.

Cell Reports, Volume *26*

Supplemental Information

Oxidative Stress Triggers Selective tRNA Retrograde Transport in Human Cells during the Integrated Stress Response

Hagen Schwenzer, Frank Jühling, Alexander Chu, Laura J. Pallett, Thomas F. Baumert, Mala Maini, and Ariberto Fassati

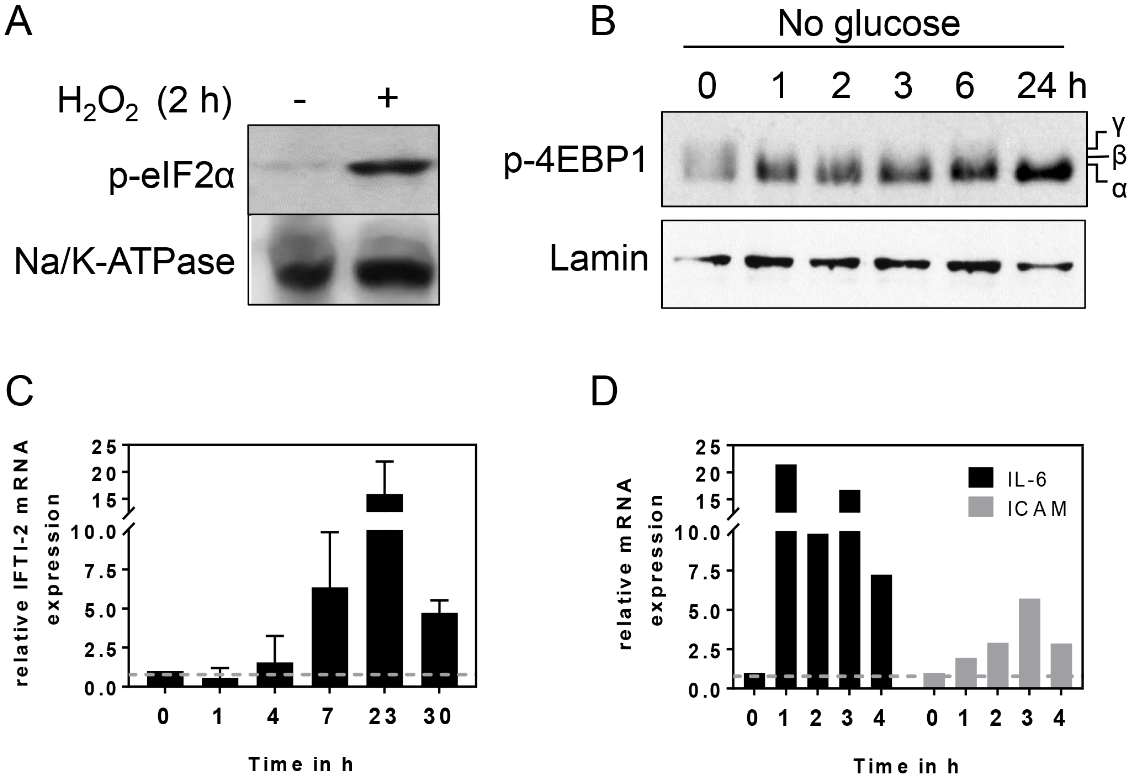

Figure S1 (related to Figure 1). Detection of cellular stress markers.

(A) Western blot to detect S51 phosphorylated eIF2α (p-eIF2α) after treatment with 5 mM H_2O_2 for 2 hours. (B) Western blot to detect changes in 4EBP1 phosphorylation at different time points in response to glucose deprivation, lamin was used as a loading control. (C) RT qPCR to measure IFTI-2 mRNA levels in response to IFNα (1.25 x10^4 U/ml). (D) Same as (C) to measure IL-6 and ICAM mRNA levels upon treatment with TNFα (1ng/ml). Values are expressed relative to GAPDH mRNA as mean ± SEM.

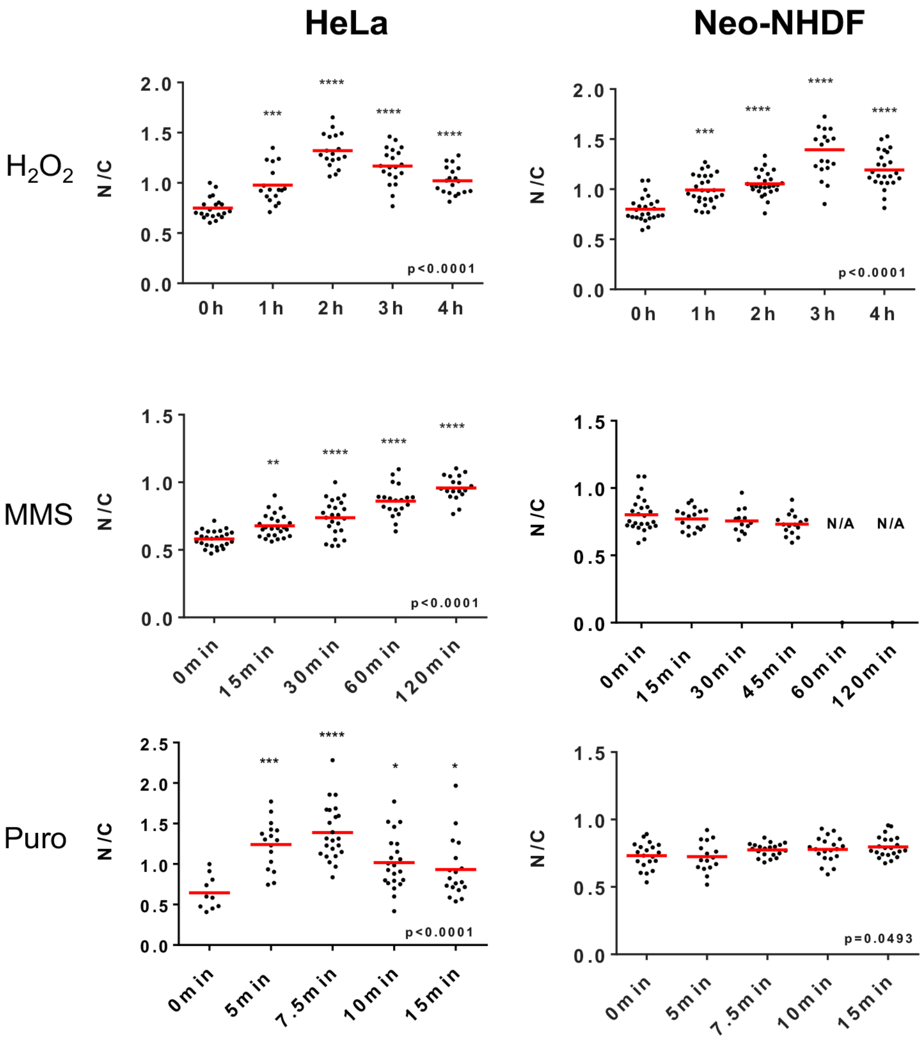

Figure S2 (related to Figure 2). Kinetics of tRNA retrograde transport in HeLa and NHDF cells. Cells were treated with 5 mM H_2O_2 or 5 mM MMS or 3 mM puromycin for the indicated time points and analyzed by tFISH and confocal microscopy. ImageJ software was used to quantify the fluorescent signal in the nucleus (N) and cytoplasm (C) and calculate the N/C ratio. Cells were counted from at least 5 randomly chosen images. Each dot corresponds to one cell. Red lines indicate the mean value. Graphs show data from one representative experiment ($n \geq 3$). One-way Anova (Dunnett's multiple comparisons test) was used to calculate statistical significance *p=0.05; **p<0.05; ****p<0.0001. N/A, not enough cells for quantification due to toxicity.

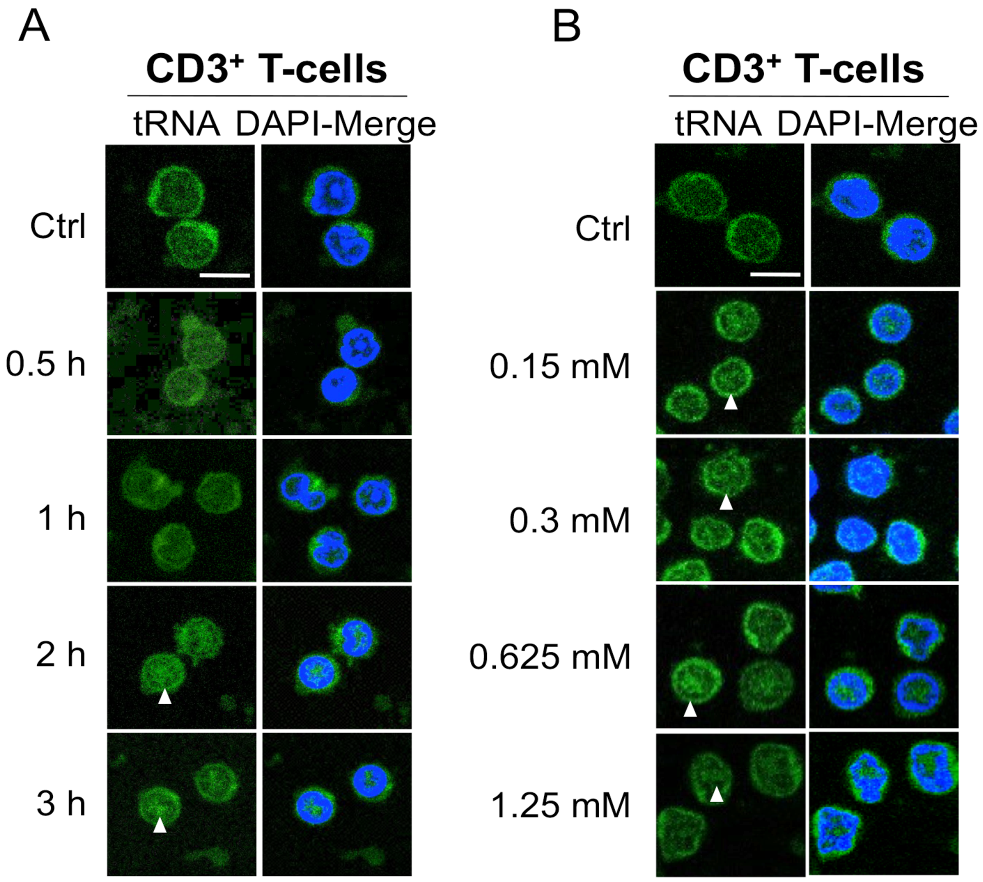

Figure S3 (related to Figure 2). Time and dose dependent induction of tRNA retrograde transport in primary $CD3^+$ T-cells.

(A) Magnetic-bead purified T-cells were exposed to 0.6 mM H_2O_2 and incubated for the indicated period of time before analysis by tFISH. Representative confocal microscopy images from one donor are shown. Similar results were obtained with a second donor. (B) T-cells were exposed for 2 hours to the indicated concentrations of H_2O_2 and analysed by tFISH and confocal microscopy. Representative confocal microscopy images are shown. Increasing concentrations of H_2O_2 led to an increase of the tRNA signal in the nucleus. The signal intensity rose until plateau was reached, afterwards the overall fluorescence intensity decreased and frequent cell death was observed. Representative images from one donor are shown. Similar results were obtained with a second donor. Arrowheads point to intra-nuclear signal. Scale bar = 10µm.

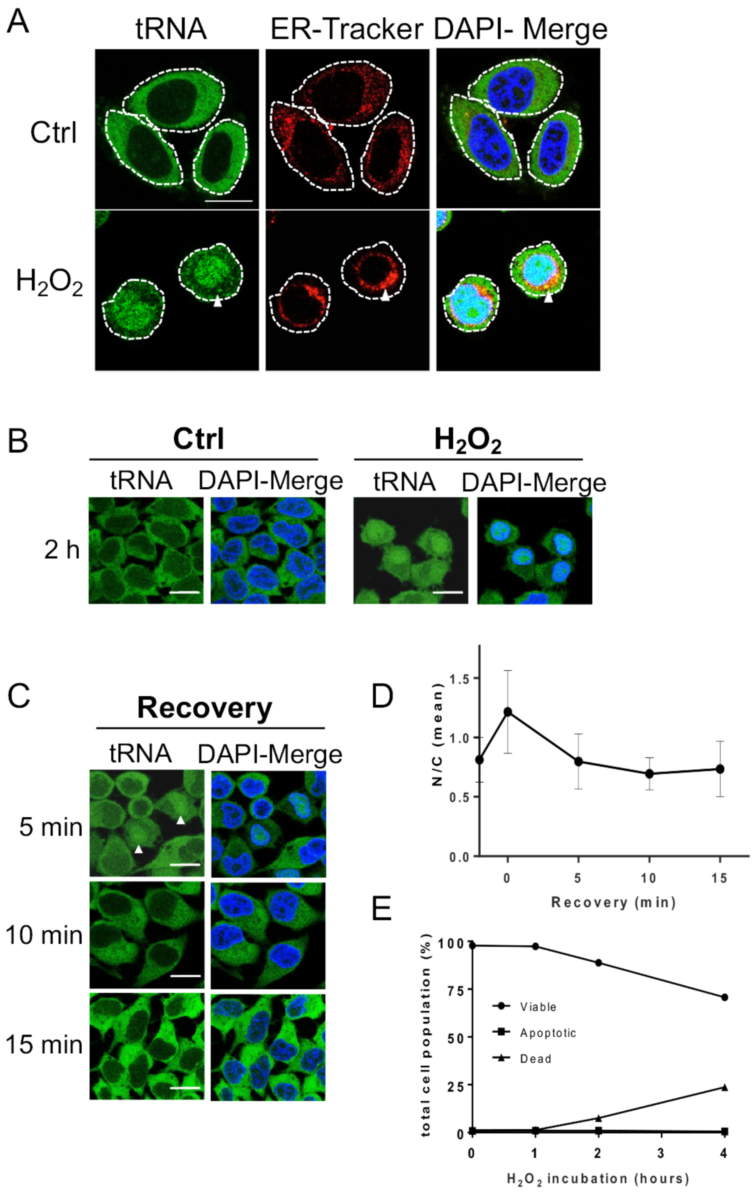

Figure S4 (related to Figure 3). tRNA retrograde transport depletes the ER and is reversible.

(A) Cells were pre-incubated with ActD for 2 hours then 5 mM H_2O_2 was added for 2 hours. Cells were incubated with ER Tracker^{TM} Red (Molecular Probes) and analysed by tFISH and confocal microscopy. Scale bar = 10µm. Arrowheads indicate the ER. (B-C) Induction of retrograde tRNA transport by H_2O_2 is reversible. Cells were exposed to 5 mM H_2O_2 for 2 hours before media was replaced without H_2O_2 (Recovery) and cells were analyzed by tFISH at the indicated time points. Representative confocal microscopy images are shown (n =3), scale bar = 20µm. Arrow heads indicate nuclei with a positive tFISH signal. (D) The tFISH fluorescent signal intensity in the nucleus and cytoplasm was quantified by ImageJ, the ratio nuclear:cytoplasmic signal (N/C) was calculated and values were plotted as a function of time. Graph shows average values ± SD from three independent experiments. (E) Flow cytometry analysis of apoptotic (annexin stain) and dead (PI stain) cells within the total population of cells treated with 5 mM H_2O_2.

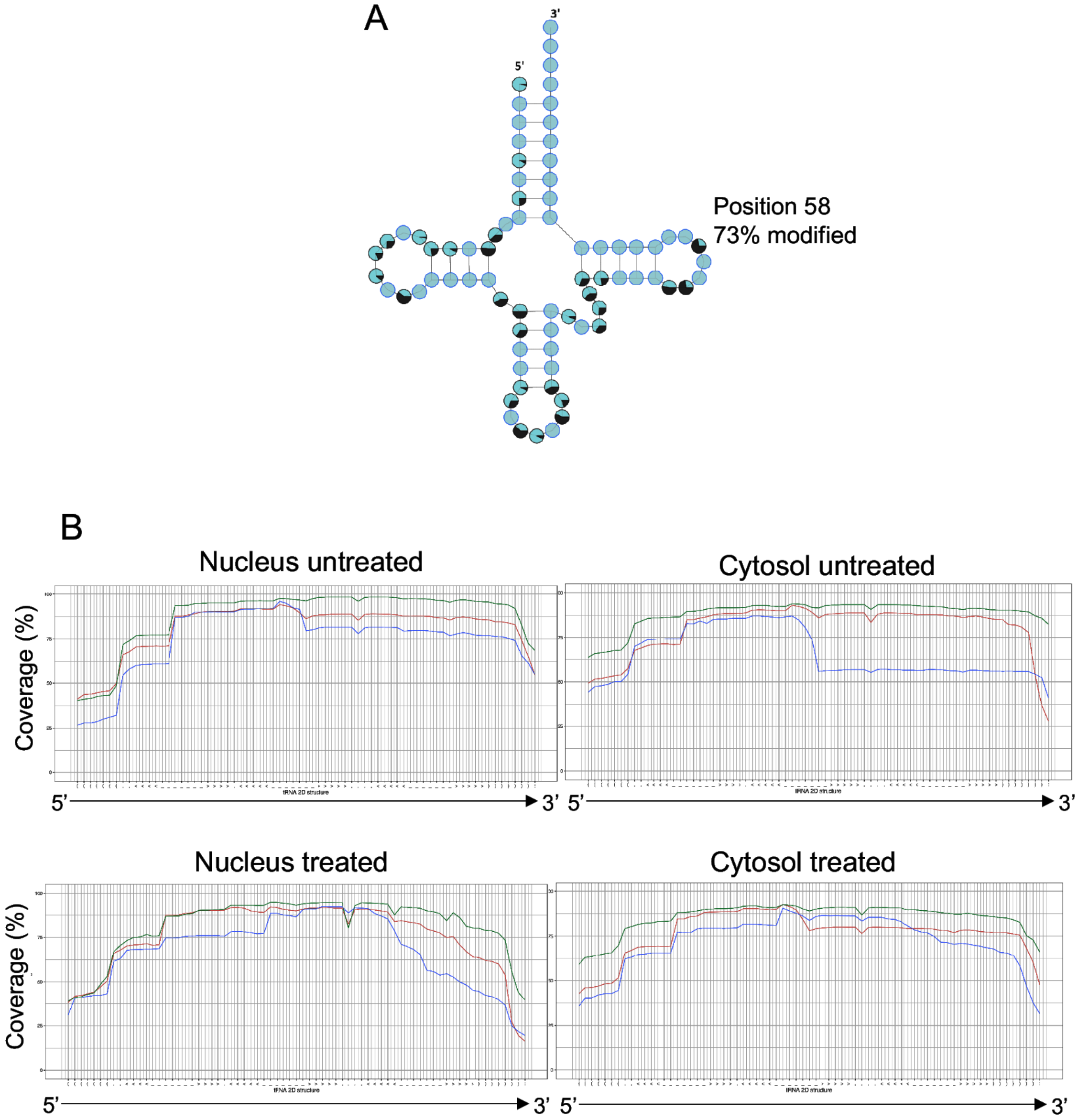

Figure S5 (related to Figure 4). Reduced 5' end coverage did not affect 3' mapping of tRNAs.

(A) Illustration of the secondary structure of the human consensus tRNA model. Each circle represents one nucleotide. Filling of circles represents a pie chart indicating the frequency of known modification at the particular position. Data has been extracted from MODOMICS (http://modomics.genesilico.pl/). (B) Alignment of sequences based on covariance model build from ~1000 tRNAs extracted from RFAM. Reads for each position have been counted and divided by total reads. Lines represent the relative coverage normalised to total tRNA reads. Each line represents one biological replicate. Treated, cells exposed for 5 mM H_2O_2 for 2 hours in the presence of ActD; untreated, cells exposed to ActD only for 2 hours.

A

	nucleus treated			cytosol treated			nucleus control			cytosol control		
**Replicates**	**1**	**2**	**3**	**1**	**2**	**3**	**1**	**2**	**3**	**1**	**2**	**3**
**total reads (in Mio)**	11.44	11.35	11.29	15.05	13.32	9.70	7.25	8.38	6.46	10.83	14.01	7.00
**mapped reads (%)**	66.76	46.93	5.04	58.34	13.07	0.33	73.94	88.60	3.21	79.89	27.78	2.26
mRNA (%)	27.61	28.94	28.95	9.22	13.56	22.66	31.95	35.34	36.66	8.79	11.27	14.89
ncRNA (%)	71.91	70.55	67.75	82.79	83.14	69.78	66.58	64.13	62.15	82.80	83.45	81.14
tRNA (%)	2.14	2.21	17.18	26.01	11.92	33.40	5.64	1.94	5.12	29.08	17.19	17.24

B

Nucleus		Cytosol	
**Ensembl ID**	**Reads ($x10^3$)**	**Ensembl ID**	**Reads ($x10^3$)**
RNU2-2P	869	MT-TV	418
SNORA51	258	CTD-2651B20.6	266
RNU5D-1	189	CTD-2651B20.7	265
SNORA2B	132	MT-TQ	261
RNU2-59P	120	MT-TN	236
SNORA28	118	RP11-473M20.16	233
SNORD14B	114	MT-TI	225
SNORA77	114	MT-TR	195
SNORA6	110	MT-TE	192
SNORA45	102	MT-TP	168

C

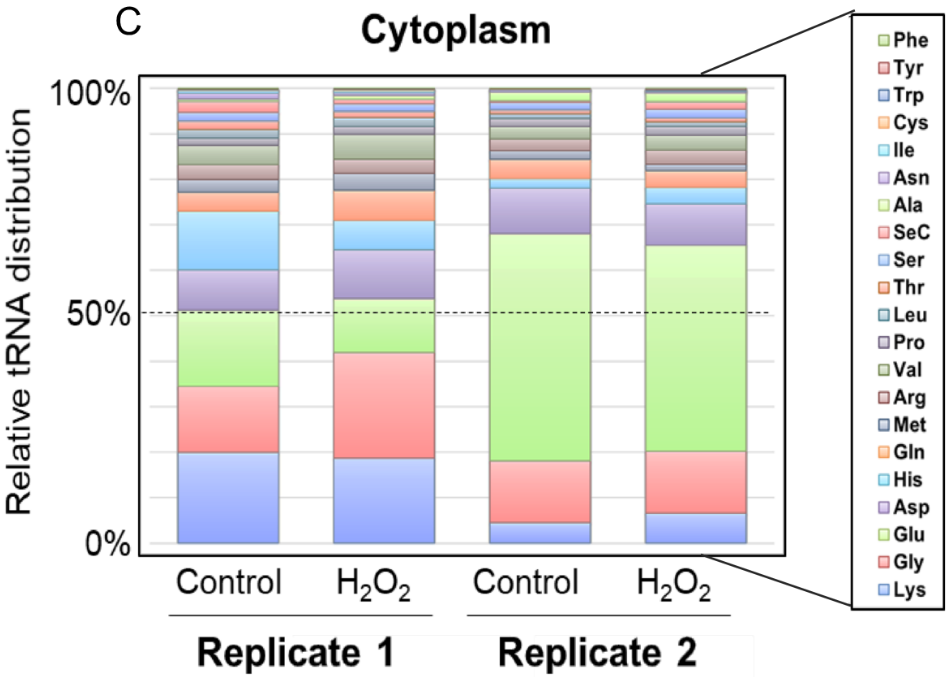

Figure S6 (related to Figure 4). Assessment of the quality of the cellular fractionation and RNAseq. HeLa cells were treated with 5 mM H_2O_2 for 2 hours in the presence of ActD and fractionated into nuclear and cytosolic fractions. RNA was extracted from each fraction and used for RNA-Seq. (A) Summary of the mapped RNA reads in each replicate and their distribution in each fraction. (B) Top 10 reads detected in the nuclear and

cytosolic fraction of one representative control replicate. Treated, cells exposed for 5 mM H_2O_2 for 2 hours in the presence of ActD; untreated, cells exposed to ActD only for 2 hours. (C) Total RNA reads were mapped to 323 unique tRNA genes and all reads mapping to the same tRNA species were counted, combined and their relative distribution calculated. tRNAs were clustered accordingly to their relative abundance in the cytoplasm. The tRNA colour code is the same for nucleus and cytoplasm. See also Tables S1 and S2.

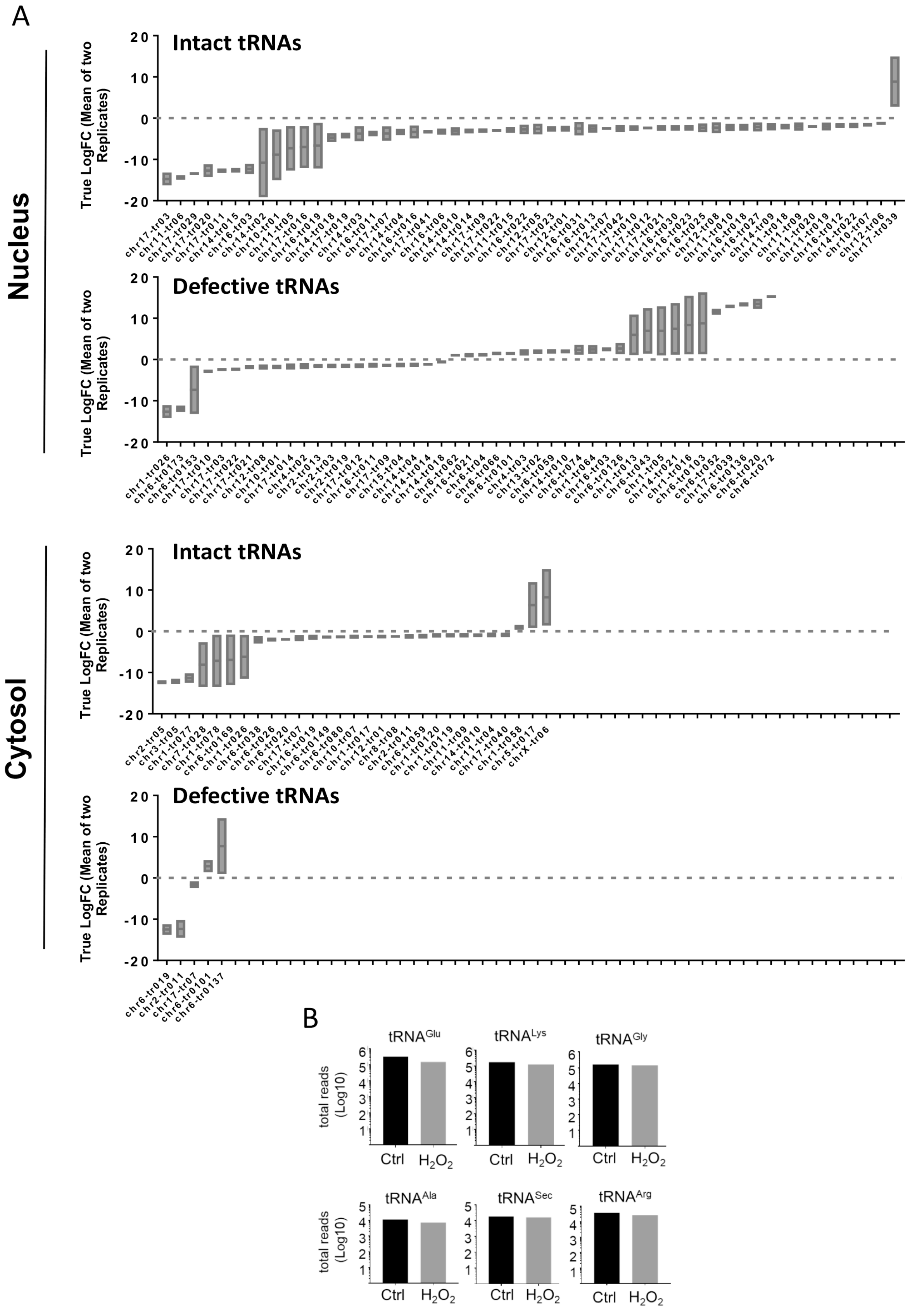

Figure S7 (related to Figure 5 and Table S4). Nuclear and cytosolic distribution of intact and 3’ end truncated

tRNA genes that show a consistent (up or down) LogFC >0.75 in each replicate (True LogFC) upon treatment with 5 mM H_2O_2 for 2 hours in the presence of ActD.

(A) tRNA-specific reads were mapped to the 3' end of the tRNAs and sorted for intact tRNAs (tRNAs with a mature 3' CCA end) and defective tRNAs (tRNA with an incomplete 3'-end). The data were plotted as average of the two replicates with min/max value. Each bar represents one tRNA gene. (see also Table S4). (B) total tRNA read counts in the nucleus or cytoplasm indicate lack of significant tRNA degradation in H_2O_2-treated relative to control cells.

Table S1

Stress	Stressor	Concentration	Time	HeLa	Neo-NHDF	CD3-T cells
**Oxidative stress**	H_2O_2	0.15, 0.3, 0.6, 1.25, 2.5, 5 (mM)	0.5, 1, 2, 3, 4 (hours)	+++	++	++
**Genotoxic stress**	Methylmethane Sulfonate (MMS)	5 mM	15, 30, 60, 120 (minutes)	+	-	++
**Translation inhibition**	Puromycin	3 mM	5, 7.5, 10, 15, 30 (minutes)	++	-	++
**Metabolic stress**	Glucose deprivation		1, 2, 3, 6, 12, 24, 48 (hours)	-	-	-
**Viral infection**	IFNα	1.15 x 10^4 U/μL	1, 2, 3, 6, 12, 24, 48 (hours)	-	-	-
**Inflammation**	TNFα	10 ng/mL	1, 2, 3, 4 (hours)	-	-	-
**Heat shock**	42 ^{0}C		15, 30, 60 (minutes)	-	ND	ND
**Low pH**	NaOH	0.1% (1M)	1, 2, 3 (hours)	-	ND	ND
**High pH**	HCl	0.1% (1M)	1, 2, 3 (hours)	-	ND	ND
**Osmotic pressure**	Sucrose	300 mM	1, 2, 3 (hours)	-	ND	ND
